# Changes in Proteome Profile of Peripheral Blood Mononuclear Cells in Chronic Chagas Disease

**DOI:** 10.1371/journal.pntd.0004490

**Published:** 2016-02-26

**Authors:** Nisha Jain Garg, Kizhake V. Soman, Maria P. Zago, Sue-Jie Koo, Heidi Spratt, Susan Stafford, Zinzi N. Blell, Shivali Gupta, Julio Nuñez Burgos, Natalia Barrientos, Allan R. Brasier, John E. Wiktorowicz

**Affiliations:** 1 Department of Microbiology and Immunology, University of Texas Medical Branch (UTMB), Galveston, Texas, United States of America; 2 Department of Pathology, UTMB, Galveston, Texas, United States of America; 3 Faculty of the Institute for Human Infections and Immunity, and Sealy Center for Vaccine Development, UTMB, Galveston, Texas, United States of America; 4 Department of Biochemistry and Molecular Biology, and the Sealy Center for Molecular Medicine, UTMB, Galveston, Texas, United States of America; 5 Instituto de Patología Experimental, CONICET-UNSa, Salta, Argentina; 6 Department of Preventive Medicine and Community Health, UTMB, Galveston, Texas, United States of America; 7 Institute for Translational Sciences, UTMB, Galveston, Texas, United States of America; 8 Servicio de Cardiología, Hospital San Bernardo, Salta, Argentina; 9 Department of Internal Medicine, UTMB, Galveston, Texas, United States of America; Hospital Universitário Professor Edgard Santos, BRAZIL

## Abstract

*Trypanosoma cruzi (Tc)* infection causes chagasic cardiomyopathy; however, why 30–40% of the patients develop clinical disease is not known. To discover the pathomechanisms in disease progression, we obtained the proteome signature of peripheral blood mononuclear cells (PBMCs) of normal healthy controls (N/H, n = 30) and subjects that were seropositive for *Tc*-specific antibodies, but were clinically asymptomatic (C/A, n = 25) or clinically symptomatic (C/S, n = 28) with cardiac involvement and left ventricular dysfunction. Protein samples were labeled with BODIPY FL-maleimide (dynamic range: > 4 orders of magnitude, detection limit: 5 f-mol) and resolved by two-dimensional gel electrophoresis (2D-GE). After normalizing the gel images, protein spots that exhibited differential abundance in any of the two groups were analyzed by mass spectrometry, and searched against UniProt human database for protein identification. We found 213 and 199 protein spots (fold change: |≥ 1.5|, p< 0.05) were differentially abundant in C/A and C/S individuals, respectively, with respect to N/H controls. Ingenuity Pathway Analysis (IPA) of PBMCs proteome dataset identified an increase in disorganization of cytoskeletal assembly and recruitment/activation and migration of immune cells in all chagasic subjects, though the invasion capacity of cells was decreased in C/S individuals. IPA predicted with high probability a decline in cell survival and free radical scavenging capacity in C/S (but not C/A) subjects. The MYC/SP1 transcription factors that regulate hypoxia and oxidative/inflammatory stress were predicted to be key targets in the context of control of Chagas disease severity. Further, MARS-modeling identified a panel of proteins that had >93% prediction success in classifying infected individuals with no disease and those with cardiac involvement and LV dysfunction. In conclusion, we have identified molecular pathways and a panel of proteins that could aid in detecting seropositive individuals at risk of developing cardiomyopathy.

## Author Summary

*C*hagasic cardiomyopathy is elicited by *Trypanosoma cruzi* infection. *T*. *cruzi* transmission is prevalent in Latin American countries, and its transmission is also noted in Mexico and Southern parts of the United States. In this manuscript, we have utilized blood samples from human subjects that were normal healthy or were infected with *T*. *cruzi* and exhibited variable symptoms of heart disease. We have employed a highly sensitive approach of protein labeling, developed a detailed proteomic map from all samples, performed comparative analysis of gel images, and identified a panel of proteins that were changed in abundance in clinically asymptomatic (C/A) and clinically symptomatic (C/S) chagasic individuals with respect to healthy controls. Functional annotation of these proteins suggested that pathologic mechanisms in disease progression would involve host’s inability to recruit immune cells, scavenge free radicals, and prevent cell death. We also describe a panel of proteins that can differentiate C/A from C/S subjects and will potentially be useful in identifying infected individuals at risk of developing clinical disease.

## Introduction

Chagasic cardiomyopathy is caused by *Trypanosoma cruzi*. According to the World Health Organization report released in 2010, ~16 million individuals are infected with *T*. *cruzi*, and >25 million people are at risk of infection in Latin America and Mexico [1]. New challenges of increased transmission are faced due to lack of sustainability of the vector control programs [2,3], migration of infected individuals to non-endemic areas (e.g. US, Canada, Europe) [4,5], and transfer of infection through blood or organ donation [6,7]. The Centers for Disease Control reports that >300,000 individuals infected with *T*. *cruzi* are currently living in the United States [8]. Several years after the initial exposure to the parasite, ~30–40% of the infected individuals develop cardiomyopathy and may progress to heart failure (reviewed in [9]). No vaccine is available for the prevention of infection [10] and the available drugs, benznidazole and nifurtimox, have exhibited no significant effects in arresting the progression of chronic cardiomyopathy [11]. Importantly, tools to assess the effectiveness of new drugs against *T*. *cruzi* infection and Chagas disease are currently not available.

We have found that *T*. *cruzi* elicits oxidative stress of inflammatory and mitochondrial origin in immune and non-immune cells; and sustained oxidative stress plays a crucial role in eliciting left ventricular dysfunction during progressive Chagas disease [9,12,13]. Our studies showed that myocardial changes in oxidant/antioxidant balance and oxidative adducts were detectable in the peripheral blood of infected mice [14] and chagasic patients [15–17]. The level of oxidative stress markers (i.e. lipid hydroperoxides) and inflammation (i.e. myeloperoxidase) increased and the level of antioxidants (e.g. manganese superoxide dismutase) decreased in both heart and peripheral blood of infected rodents with progressive disease [14]. These studies, thus, support the notion that peripheral blood cells provide a suitable tissue for delineating the pathways that are deregulated during the chronic development of chagasic cardiomyopathy.

In this study, we have employed a quantitative saturation fluorescence labeling approach for the detection of the differential protein signature of peripheral blood mononuclear cells (PBMCs) in *T*. *cruzi*-infected subjects. All enrolled subjects were assessed by electrocardiography and transthoracic echocardiography and characterized for the severity of cardiac disturbances. We employed a thiol-labeling maleimide dye under saturating conditions that exhibits stable, specific, quantitative labeling of cysteine residues in conjunction with two-dimension electrophoresis and mass spectrometry for developing the PBMCs’ proteome of chagasic patients. Up to 92% of the human proteins contain at least one cysteine residue [18], and thus can be detected using the thiol-labeling maleimide dye. Our findings provide clues to the molecular pathways that may be disturbed with development of chronic Chagas disease. We discuss a panel of proteins that could potentially be useful in classifying the disease state and identifying asymptomatic individuals at risk of developing clinical disease.

## Materials and Methods

### Human samples

#### Ethics statement

This study was conducted under a human subjects study protocol approved by the institutional review board at the University of Texas Medical Branch at Galveston (IRB04-257) and the ethics committee at the Universidad Nacional de Salta in Salta, Argentina. Blood samples were obtained from individuals living in Salta Argentina. A written informed consent was obtained from all individuals, and samples were decoded and de-identified before they were provided for research purposes. Subjects with co-morbid diseases, e.g., HIV/AIDS, *Leishmaniasis*, autoimmune disorders, or chronic hepatic, renal or pulmonary disease were excluded from the study [15]. Please see Table 1 for patients' demographic data.

**Table 1:** Characterization of the subjects included in the study.

Clinical characterization	Subjects numbers	Age in years (mean ± SD)	Sex males (%)
*Seropositive for T*. *cruzi-specific antibodies*			
Chagasic, clinically-asymptomatic	n = 25	49.8 ± 9.2	14 (46.6%)
Chagasic, clinically-symptomatic	n = 28	53.1 ± 10.6	16 (53.3%)
*Seronegative for T*. *cruzi-specific antibodies*			
Normal healthy, no disease	n = 30	39 ± 16.2	15 (50%)

Subjects were screened for *T*. *cruzi*-specific antibodies by Wiener Chagatest-ELISA and Wiener Chagastest-HAI kits. Clinical exam included physical exam, electrocardiography and echocardiography. Seropositive individuals with no to minor echocardiography abnormalities, no left ventricular dilatations, preserved systolic function (ejection fraction: 55–70%) were considered clinically-asymptomatic. Seropositive individuals with varying degree of heart involvement evidenced by systolic dysfunction (ejection fraction: <55%), left ventricular dilatation (diastolic diameter ≥57 mm), and/or potential signs of congestive heart failure were classified as clinically-symptomatic.

All sera samples were analyzed for *T*. *cruzi-*specific antibodies by using the Chagatest-ELISA and Chagatest-HAI kits, following the instructions provided by the manufacturer (Wiener, Rosario, Argentina). For ELISA, 96-well plates were coated with *T*. *cruzi* recombinant proteins provided in the kit, and then plates were incubated with sera samples (1:20 dilution) and HRP-conjugated secondary antibody. Color was developed with TMB substrate, and change in absorbance recorded at 450 nm. For indirect heamagglutination test, several 4-fold dilutions of the sera samples (25-μl/well) were added in duplicate to 96-well plates. Then, red blood cells sensitized with *T*. *cruzi* cytoplasmic and membrane antigens were added to the 96 well plates, and *Tc*-specific antibodies dependent agglutination of red blood cells was monitored by light microscopy. The titer was defined as the highest serum dilution presenting agglutination (positive ≥ 1:16 dilution). Samples that were found to be positive by both tests were identified as seropositive [15,19].

All individuals were provided a routine physical exam, and subjective frequency or severity of exertional dyspnea noted. Electrocardiography (ECG, 12-lead at rest and 3-lead with exercise) was performed to assess the electrical activity of the heart as previously described [15]. Transthoracic echocardiography was performed to assess the left ventricular (LV) function at diastole and systole [19]. Based upon clinical data, individuals were categorized as normal healthy (N/H) if they exhibited no history or clinical symptoms of heart disease. Seropositive individuals were grouped as clinically asymptomatic (C/A) when they exhibited none to minor ECG abnormalities, no left ventricular dilatations, and normal ejection fraction (EF) of 55–70%. Seropositive individuals were categorized as clinically symptomatic (C/S) when they displayed varying degree of ECG abnormalities, systolic dysfunction (EF: <55%), left ventricular dilatation (diastolic diameter ≥57 mm), and/or potential signs of congestive heart failure [15,19].

### PBMC isolation, BODIPY labeling and two-dimension electrophoresis

All chemicals and reagents were of molecular grade (>99.5% purity). BD Vacutainer CPT Cell Preparation Tubes (heparinized) containing 8 ml whole blood samples were centrifuged following manufacturer’s instruction. The FICOLL Hypaque™ density gradient was employed to enrich the PBMC fraction, and the latter was pelleted by centrifugation at room temperature at 400 x g for 10 min. The PBMC pellets were suspended in 1 ml of hypotonic buffer to lyse contaminating red blood cells, and 9 ml of complete RPMI-1640 medium / 10% fetal bovine serum (Invitrogen) added. After centrifugation as above, final cell pellets consisting of 8-10-million PBMCs were stored at -80°C.

PBMC pellets from individual study subjects were lysed in 7 M urea, 2 M thiourea, 2% CHAPS, and 50 mM Tris (pH 7.5), containing benzonase nuclease (300-units/ml), as described previously [20,21]. Protein concentrations were determined by using a Pierce Modified Lowry Protein Assay Kit, and cysteine (cysteic acid) levels in all samples were determined by using an Amino Acid Analyzer (Model L8800, Hitachi High Technologies America, Pleasanton, CA) [20]. Samples were incubated for 1 h with 6 mM ascorbate (Asc) to ensure all cysteine residues were reduced and available for dye-binding, dialyzed against urea buffer to remove excess ascorbate, and then labeled with BODIPY FL *N*- (2-aminoethyl) maleimide (BD from Life Technologies, Grand Island, NY) at 60-fold excess to cysteine [21]. The mixtures were incubated for 2 h; the reactions were stopped with a 10-fold molar excess of 2-mercaptoethanol over dye. All incubations were carried out at room temperature in the dark in 200 μl reaction volume [20,21].

BD-labeled PBMC lysates (100 μg protein) were separated by 2-dimension electrophoresis (2DE), employing an IPGphor multiple sample isoelectric focusing (IEF) device (GE Healthcare) in the first dimension, and the Criterion Dodeca cell (Bio-Rad) in the second dimension, as we have described previously [22,23]. Briefly, samples were loaded on to 11 cm dehydrated precast immobilized pH gradient (IPG) strips (GE Healthcare), and strips were rehydrated overnight. IEF was performed at 20°C with the following parameters: 50 V, 11 h; 250 V, 1 h; 500 V, 1 h; 1,000 V, 1 h; 8,000 V, 2 h; 8,000 V, 48,000 V/h. The IPG strips were then incubated in 10 ml of equilibration buffer (6 M urea, 2% sodium dodecyl sulfate (SDS), 50 mM Tris-HCl, pH 8.8, 20% glycerol) for 30 min at 22°C, and electrophoresis was performed at 150 V for 2.25 h, 4°C using precast 8–16% polyacrylamide gels in Tris-glycine-SDS buffer (25 mM Tris-HCl, 192 mM glycine, 0.1% SDS, pH 8.3) [22,23].

### Image processing and analysis

Gels were fixed in 20% methanol / 7% acetic acid / 10% acetonitrile for 1 h and washed with 20% ethanol / 10% acetonitrile to reduce background. Gel images were acquired at 100 μm resolution using the Typhoon Trio Variable Mode Imager (GE Healthcare) to quantify BD-labeled proteins (Ex_488 nm_ / Em_520 nm_). Up to 92% of the human proteins contain at least one cysteine residue [18]. The Totallab SameSpots software (formerly Nonlinear Dynamics Ltd. Newcastle, UK) selects one reference gel according to several criteria, including quality and number of spots with the intent on selecting the gel that best represents all the gels. The reference gel containing the most common features was selected from the pool of gels of the N/H samples, and all data were then derived by comparison to the N/H reference gel. To ensure that the maximum numbers of proteins were detected, the reference gel was also stained with SyproRuby (Life Technologies Grand Island, NY) that binds all proteins irrespective of presence or absence of cysteine amino acid, and gel image was acquired at Ex_488nm_/Em_560nm_. The exposure time for both dyes (BD and SyproRuby) was adjusted to achieve a value of ~55,000–63,000 pixel intensity (16-bit saturation) from the most intense protein spots on the gel [22,23].

In total, 83 BD-stained 2D gels representing 30, 25, and 28 samples from N/H, C/A, and C/S subjects, respectively, were scanned and analyzed with the Totallab SameSpots software. After manual and automated pixel-to-pixel alignment, the program performed automatic spot detection on all images. The SyproRuby stained reference gel was used to define spot boundaries; however, the gel images taken under the BD-specific filters were used to obtain the quantitative spot data. This strategy ensures that spot numbers and outlines were identical across all gels in the experiment, eliminating problems with unmatched spots as well as ensuring that the greatest number of protein spots and their spot volumes were accurately detected and quantified [23]. Protein spot abundance ratios were calculated from normalized spot volumes from affected samples versus the matched normal spot volumes (Δ protein abundance = Asc^+^chagasic/Asc^+^ N/H controls). Spot volumes were normalized for each sample using a software-calculated bias value assuming that the great majority of spot volumes did not change in abundance (log (abundance ratio) = 0). The scatter of the log (abundance ratios) for each spot in a gel (sample) is distributed around some mean value that represents the systematic factors that govern the experimental variation. Thus, a gain factor is calculated to adjust the mean spot ratios of a given gel to 0 (log (abundance ratio) = 0) and applied to each spot volume [23].

For the purpose of selecting differentially abundant protein spots for mass spectrometry, normalized spot volumes were subjected to statistical analysis using in-built tools in Totallab SameSpots software. Spot volumes were log2 transformed and spot-wise standard deviation, arithmetic mean, and coefficient of variation (CoV) values of the standard abundance values were calculated for each spot [24]. Student’s *t*-tests with Welch’s correction for unequal variances were used to test for differential protein expression between N/H controls and either C/A or C/S chagasic subjects. Benjamini-Hochberg multiple hypothesis testing correction was applied to account for the false discovery rate and significance was accepted at p<0.05. The protein spots identified to be differentially abundant (p< 0.05) in at least one of the groups were submitted for mass spectrometry identification.

### Matrix assisted laser desorption ionization-time of flight (MALDI-TOF)/mass spectrometry (MS) for protein identification

Selected spots on the 2D gels that exhibited significant differential prevalence (p≤0.05) in at least one of the group were picked robotically (ProPick II, Digilab, Ann Arbor, MI), and trypsin digested as described by us [19,25]. In brief, gel spots were incubated at 37°C for 30 min in 50 mM NH_4_HCO_3_, dehydrated twice for 5 min each in 100-μl acetonitrile, dried, and proteins were digested in-gel at 37°C overnight with 10 μl of trypsin solution (1% trypsin in 25 mM ammonium bicarbonate). Peptide mixtures (1-μl) were directly spotted onto a MALDI-TOF MS/MS target plate with 1 μl of alpha-cyano-4-hydroxycinnamic acid matrix solution (5 mg/ml in 50% acetonitrile), and analyzed using a MALDI-TOF/TOF AB Sciex TOF/TOF 5800 Proteomics Analyzer (Framingham, MA). The Applied Biosystems software package included the 4000 Series Explorer (v.3.6 RC1) with Oracle Database Schema (v.3.19.0) and Data Version (3.80.0) to acquire and analyze MS and MS/MS spectral data. The instrument was operated in a positive ion reflectron mode with the focus mass set at 1700 Da (mass range: 850–3000 Da). For MS data, 1000–2000 laser shots were acquired and averaged from each protein spot. Automatic external calibration was performed by using a peptide mixture with the reference masses 904.468, 1296.685, 1570.677, and 2465.199. Following MALDI MS analysis, MALDI MS/MS was performed on several (5–10) abundant ions from each protein spot. A 1-kV positive ion MS/MS method was used to acquire data under post-source decay (PSD) conditions. The instrument precursor selection window was +/- 3 Da. Automatic external calibration was performed by using reference fragment masses 175.120, 480.257, 684.347, 1056.475, and 1441.635 (from precursor mass 1570.700) [19,25].

For protein identification, the MS and MS/MS spectral data were searched against the UniProt human protein database (last accessed: March 25, 2013; 87,656 sequences; 35,208,664 residues) by using a AB Sciex GPS Explorer (v.3.6) software in conjunction with MASCOT (v.2.2.07) as described previously [19]. The protein match probabilities were determined by using expectation values and/or MASCOT protein scores. The MS peak filtering included the following parameters: a mass range of 800 Da to 3000 Da, minimum S/N filter = 10, mass exclusion list tolerance = 0.5 Da, and mass exclusion list for some trypsin and keratin-containing compounds included masses (Da) 842.51, 870.45, 1045.56, 1179.60, 1277.71, 1475.79, and 2211.1. The MS/MS peak filtering included the following parameters: minimum S/N filter = 10, maximum missed cleavages = 1, fixed modification of carbamidomethyl (C), variable modifications due to oxidation (M), precursor tolerance = 0.2 Da, MS/MS fragment tolerance = 0.3 Da, mass = monoisotopic, and peptide charges = +1. The significance of a protein match, based on the peptide mass fingerprint (PMF) in the MS and the MS/MS data from several precursor ions, is presented as expectation values (*p*<0.05). To confirm the identified proteins were of human and not of parasite origin, we also performed a similar search against NCBI non-redundant protein database consisting of *T*. *cruzi* sequences.

In cases where abundance was ≥ |2| but protein IDs were ambiguous (protein scores <62), the digested proteins were submitted for analysis by LTQ OrbiTrap Velos (ThermoFisher, Waltham, MA).

### Functional analysis, and multivariate adaptive regression splines (MARS) modeling

We used the Ingenuity Pathways Analysis (IPA) web-based application (Ingenuity Systems, Redwood city, CA) to assess the biological meaning in the proteome datasets. IPA retrieves biological information from the literature—such as gene name, sub-cellular location, tissue specificity, function, and association with disease—and then integrates the identified proteins into networks and signaling pathways with biological meaning and significance [26]. An “e-value” was calculated by estimating the probability of a random set of proteins having a frequency of annotation for that term greater than the frequency obtained in the real set, and a significance threshold of 10^−3^ was used to identify significant molecular functions and biological processes [19]. With these parameters, we were able to highlight the most informative and significantly over-represented gene ontology terms in the dataset [19,27].

For MARS modeling, normalized spot volumes for all spots from 83 gels were exported from SameSpots in to Excel, and analyzed by using R and SPSS ver.20 software. For modeling the disease state specific response, a stringent cut-off was applied; differentially abundant protein spots were first screened by t test/Welch’s correction and then Benjamini-Hochberg test was employed at p<0.001 (≥І1.5І fold change). MARS was employed to model changes in multiple variables for distinguishing between infection and disease status [24]. We used 10-fold cross-validation and 80% (training)/20% (testing) approaches to predict the protein spots that can distinguish N/H from C/A and C/S subjects. The sensitivity and specificity of the identified models were validated by receiver operator characteristics (ROC) curves.

## Results

### Chagas subjects exhibit disease state-specific PBMC proteome signature

All protein extracts were analyzed for cysteine content by amino acid analysis and labeled with uncharged BODIPY FL-maleimide (BD, dye-to-protein thiol ratio > 60:1). The saturation fluorescence labeling with BD provided no non-specific labeling, had no effect on the isoelectric point and mobilities of the proteins, and provided a linear dynamic range of over four orders of magnitude in identifying the protein spots (detection limit: 5 f mol protein in a gel spot at a signal-to-noise ratio of 2:1), as we have also noted in a previous study [23].

**Fig 1:**
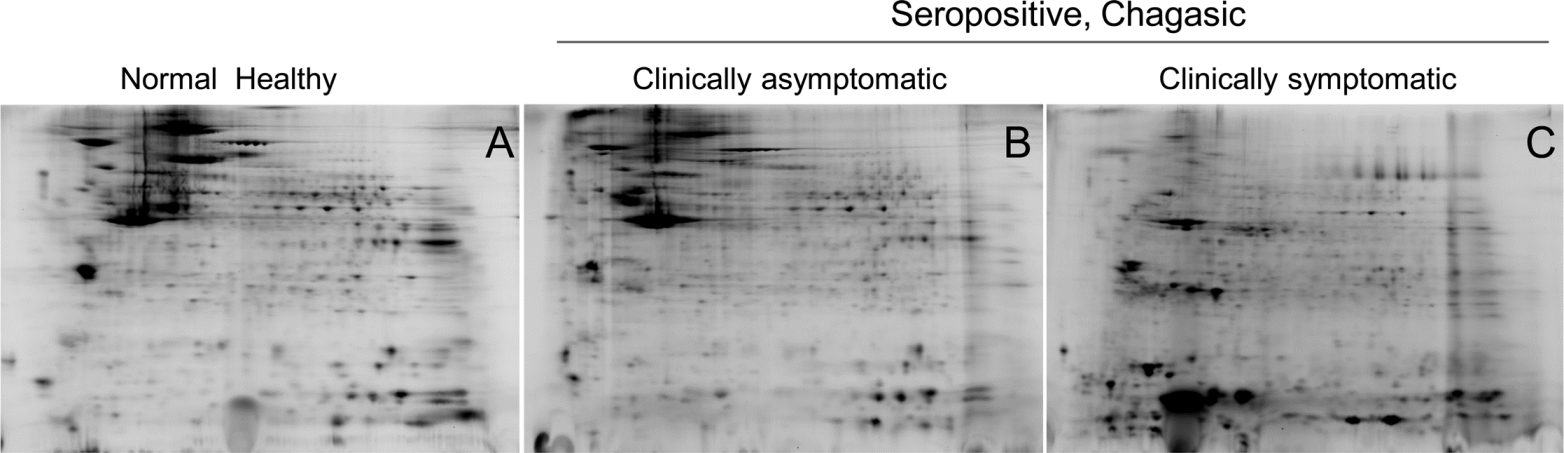
Two-dimensional gel images of protein spots in PBMCs of chagasic patients and healthy controls. PBMCs from seropositive chagasic subjects categorized as clinically asymptomatic (C/A, n = 25) and clinically symptomatic (C/S, n = 28), and normal healthy (N/H, n = 30) controls were reduced in presence of ascorbate, and labeled with BODIPY FL *N*- (2-aminoethyl) maleimide that covalently labels cysteine residues. The BD-labeled protein samples were separated in the 1^st^-dimension by isoelectric focusing on 11 cm linear pH 4–7 immobilized pH gradient strips, and in the 2^nd^-dimension by sodium dodecyl sulfate polyacrylamide gel electrophoresis (SDS-PAGE) on an 8–16% gradient gel. Gel images were obtained at 100 μm resolution using the Typhoon Trio Variable Mode Imager (GE Healthcare) to quantify BD-labeled proteins (Ex_488 nm_ / Em_520±15 nm_). Shown are representative gel images of PBMCs from N/H **(A)**, C/A **(B)** and C/S **(C)** subjects.

PBMC lysates of the normal healthy (N/H) controls (n=30), and of seropositive, clinically asymptomatic (C/A, n=25) and seropositive, clinically symptomatic (C/S, n=28) individuals were resolved by 2D-GE. The representative 2D gel images for these groups are shown in Fig 1A–1C. All protein spots were within the relative molecular sizes 10 to 250 kDa.

**Fig 2:**
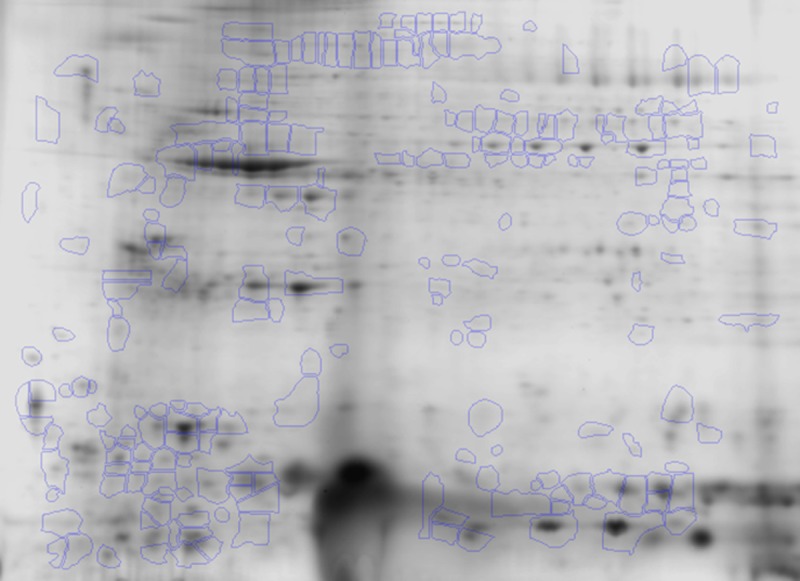
Identification of differentially abundant protein spots in chagasic PBMCs. Of all the protein spots identified by 2-dimension electrophoresis, ratiometric calculation from BODIPY-fluorescence units in Asc+ aliquots (normal versus experimental) was conducted for quantifying differential abundance of proteins (Δ protein abundance = Asc^+^chagasic/Asc^+^ controls). The fold-change in protein spots in all gels were log transformed and submitted to statistical analysis as described in Materials and Methods. Protein spots that exhibited significant change in abundance in chagasic groups with respect to controls (p<0.05) are marked, and were submitted to MALDI-TOF MS analysis for protein identification (listed in Table 2).

**Fig 3:**
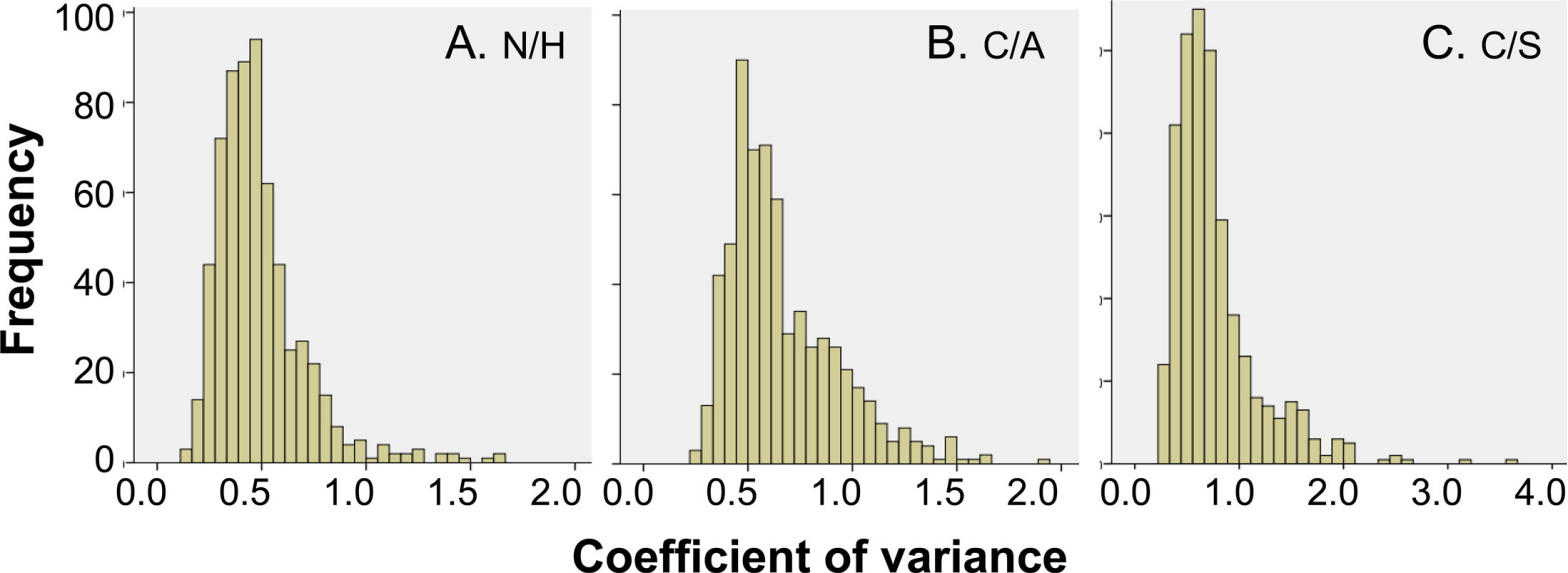
Distribution of variation in abundance of protein spots. Shown are distribution of coefficient of variation (CoV) values of the standard abundance values for each protein spot identified in PBMCs of N/H controls (30 gels, panel A) and C/A (25 gels, panel B) and C/S (28 gels, panel C) subjects.

All of the 2D gel images were assessed for quality control by SameSpots software, and then aligned both manually and automatically against the reference gel (Fig 2), chosen from the entire set of gel images by the software. The fluorescence intensity of the protein spots was normalized using a bias factor calculated assuming most spots did not change across the experiment. The log2 transformed abundance values for each protein spot on 2D gels were utilized to calculate the mean coefficient of variation (CoV) values (Fig 3) for the biological replicates. These data showed the mean CoV values were 49 ± 21.7%, 67 ± 26.4%, and 77 ± 41.3%, for N/H, C/A and C/S groups, respectively (Fig 3A–3C). Up to 75% of the spots in all groups did not exceed the CoV value of 80% indicating that most of the protein abundances are quite stable in the different groups. Protein spots exceeding a CoV of 100% were largely noted in chagasic subjects, indicating a changing and variable protein expression pattern with disease progression.

**Fig 4:**
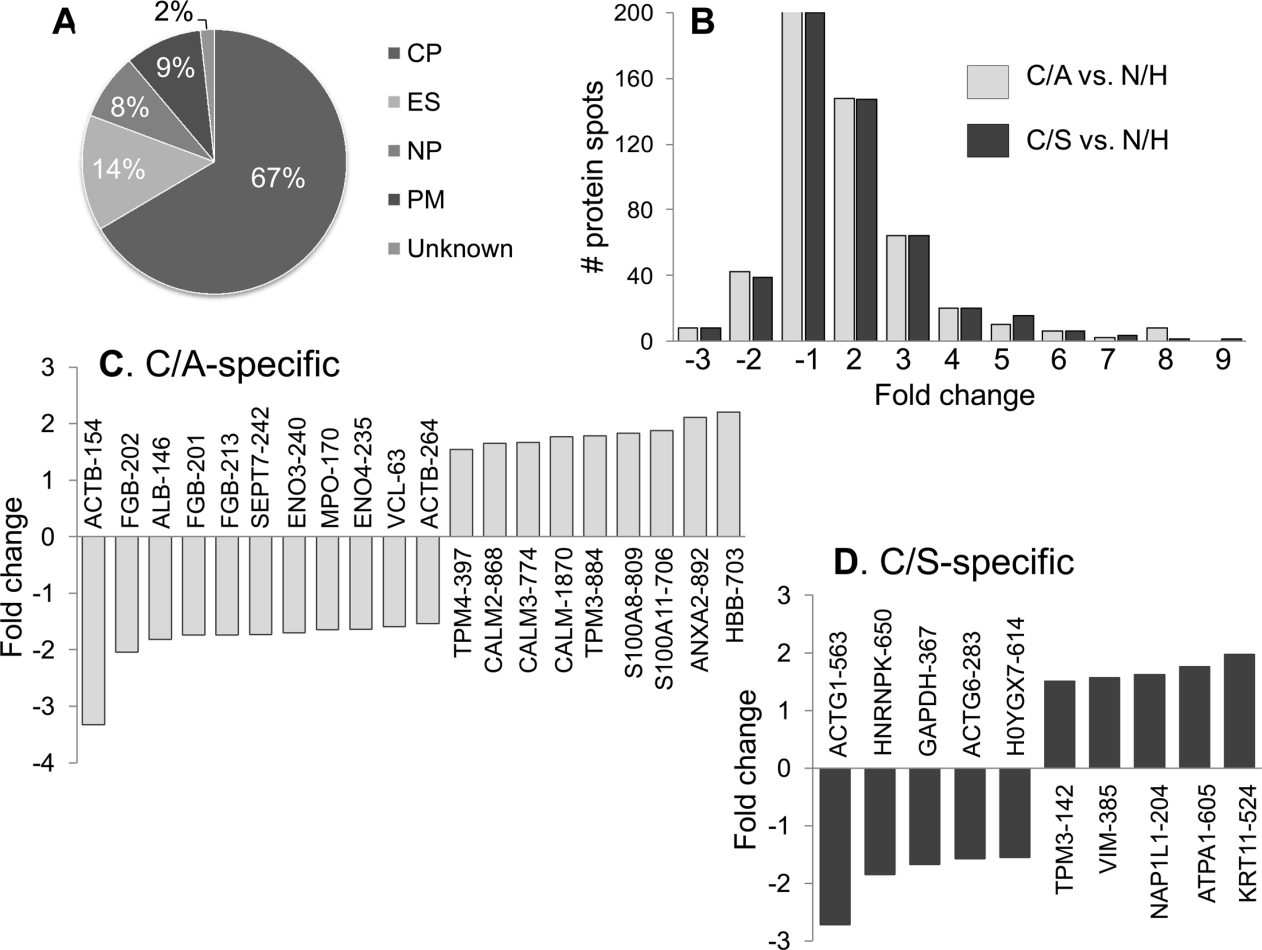
Disease specific proteome signature in chagasic subjects. **(A)** Ontological classification of differentially regulated proteins in terms of cellular localization was performed by Ingenuity Pathway Analysis. The compositions of the protein categories are presented as percentages of all individually identified proteins. **(B)** Shown are the frequency of protein spots that were changed in abundance in clinically-asymptomatic (C/A) and clinically-asymptomatic (C/S) chagasic subjects with respect to normal/healthy (N/H) controls (p<0.05). **(C&D)** Bar graphs show the protein molecules that were uniquely changed in abundance in C/A *(C)* and C/S *(D)* subjects. Data are plotted as fold change in comparison to N/H controls.

For the purpose of selecting protein spots for identification by mass spectrometry, the protein spot datasets were analyzed in pair-wise manner by *t* test with Welch's correction that accounts for unequal variances. This analysis yielded 315 (162 up-regulated, 153 down-regulated, p<0.05) and 348 (180 up-regulated, 168 down-regulated, p<0.05) differentially abundant protein spots in seropositive subjects with no disease and those with LV dysfunction, respectively. These datasets were then submitted to Benjamini-Hochberg multiple hypothesis testing correction to adjust the false discovery rate, and the differentially abundant protein spots (fold change: |≥1.5|, p<0.05 with B-H correction) were submitted for MALDI-TOF/TOF analysis. Homology searches were conducted against the UniProt’s human proteome database for protein identification [19]. A total of 213 protein spots (102 up-regulated, 111 down-regulated, fold change: |≥1.5|) in seropositive/clinically-asymptomatic subjects; and 199 protein spots (97 up-regulated, 102 down-regulated, fold change: |≥1.5|) in seropositive subjects with LV dysfunction were found to be differentially expressed with respect to normal controls, and identified by mass spectrometry (Table 2). These proteins were predicted to be localized in cytoplasm (67%), extracellular space (14%), nucleus (8%), or plasma membrane (9%) (Fig 4A). The changes in abundance frequency of the identified proteins ranged from > -3-fold to >9-fold in chagasic subjects (Fig 4B). A majority of the identified protein spots were differentially abundant in all chagasic subjects though the extent of change in expression was more pronounced in seropositive subjects with LV dysfunction. When we compared the differential abundance of proteins in seropositive C/A versus C/S subjects, we noted 20 and 10 protein spots that were uniquely changed in abundance in clinically-asymptomatic (Fig 4C) and clinically-symptomatic subjects (Fig 4D), respectively, and were relevant to disease state.

**Table 2:** Proteome profile of PBMC proteins in human patients with *T*. *cruzi* infection and Chagas disease.

Protein name	Gene name	Accession No.	Spot No.	pI	MW (kDa)	Protein score	E value	C/A-vs-N/H	C/S-vs-N/H	Localization
Actin, alpha 1, skeletal muscle	ACTA1	Q5T8M8	890	4.94	16	124	3.15E-08	2.63	2.49	CP
Actin, alpha 1, skeletal muscle	ACTA1	Q5T8M7	121	5.41	68	227	1.58E-18	-2.68	-2.63	CP
Actin, cytoplasmic 1	ACTB	C9JUM1	769	5.38	14	44	3.15E+00	3.52	5.26	CP
	ACTB	C9JUM1	679	7.66	13	103	3.97E-06	2.42	2.61	CP
Actin, cytoplasmic 1	ACTB	P60709	629	4.35	15	65	2.51E-02	2.54	3.67	CP
			303	4.54	38	91	6.29E-05	-1.75	-1.78	
			657	4.79	15	108	1.26E-06	4.55	4.91	
			642	4.21	15	139	9.98E-10	2.28	2.73	
			621	4.59	16	153	3.97E-11	2.63	2.77	
			896	4.29	14	198	1.26E-15	7.99	8.88	
			335	5.55	35	225	2.51E-18	2.31	2.44	
			676	4.43	13	225	2.51E-18	2.90	2.88	
			476	4.74	21	226	1.99E-18	1.90	2.27	
			675	4.29	13	232	5.00E-19	3.85	4.04	
			665	4.45	14	233	3.97E-19	4.59	4.42	
			897	4.3	14	242	5.00E-20	7.01	7.65	
			677	4.61	13	244	3.15E-20	2.35	2.38	
			321	5.32	36	247	1.58E-20	1.81	1.72	
			662	4.65	14	263	3.97E-22	4.85	4.92	
			232	5.76	45	281	6.29E-24	-1.78	-1.72	
			238	5.5	45	294	3.15E-25	-2.23	-2.28	
			101	5.41	83	297	1.58E-25	-1.84	-1.90	
			268	6.69	41	298	1.26E-25	-1.89	-1.61	
			254	7.25	42	318	1.26E-27	-2.35	-2.23	
			479	5.36	21	334	3.15E-29	2.14	2.46	
			264	4.86	41	336	1.99E-29	-1.54	ND	
			781	4.61	27	342	5.00E-30	-2.52	-2.10	
			756	5.29	45	343	3.97E-30	-2.86	-3.02	
			69	5.39	99	347	1.58E-30	-2.26	-2.06	
			180	5.14	54	353	3.97E-31	-1.91	-2.76	
			185	5.5	53	365	3.97E-32	-1.96	-3.00	
			270	4.98	41	383	3.97E-34	-1.78	-1.50	
			244	5.1	44	390	7.92E-35	-2.58	-2.38	
			154	5.34	59	391	6.29E-35	-3.33	ND	
			184	5.29	53	394	3.15E-35	-3.05	-1.64	
			763	5.73	20	416	1.99E-37	2.43	2.23	
			275	5.19	40	418	1.26E-37	-1.68	-1.68	
			779	4.63	28	428	1.26E-38	-2.32	-1.92	
			337	5.81	34	456	1.99E-41	1.85	2.09	
Actin, cytoplasmic 2	ACTG1	I3L1U9	563	5.99	18	61	6.20E-02	ND	-2.72	CP
Actin, cytoplasmic 2	ACTG1	P63261	255	7.46	42	55	2.51E-01	-2.31	-2.28	CP
			613	4.61	16	61	6.29E-02	4.67	3.96	
			610	4.48	16	103	3.97E-06	2.40	2.68	
			512	5.5	20	150	7.92E-11	1.64	1.98	
			269	6.91	41	170	7.92E-13	-2.33	-2.00	
			283	5.86	39	170	7.92E-13	ND	-1.57	
			762	4.91	16	221	6.29E-18	2.03	2.20	
			659	4.47	14	255	2.51E-21	5.17	5.24	
Actin, cytoplasmic 2	ACTG1	P60709	267	6.41	41	290	6.29E-24	1.30	1.69	CP
			118	5.55	69	269	9.98E-23	-1.82	-1.90	
Actin, cytoplasmic 2	ACTG1	K7EM38	891	5.03	15	113	3.97E-07	5.01	4.22	CP
			623	5.16	16	130	7.92E-09	3.55	3.05	
			759	4.82	15	263	3.97E-22	4.82	4.68	
			664	7.01	14	41	6.29E+00	2.07	2.33	
Albumin	ALB	P02768	146	6.78	60	116	1.99E-07	-1.82	ND	ES
			79	6.48	96	190	7.92E-15	-3.06	-3.36	
Alpha-actinin-1	ACTN1	H0YJ11	588	3.88	17	50	7.92E-01	2.64	2.19	CP
Alpha-enolase	ENO1	P06733	233	7.09	45	128	1.26E-08	-1.98	-1.59	CP
			256	7.56	42	166	1.99E-12	-2.87	-2.16	
			240	7.58	45	347	1.58E-30	-1.70	ND	
			234	7.24	45	363	3.97E-32	-1.75	-1.65	
			247	8.41	44	423	3.97E-38	-1.86	-2.01	
Annexin A1	ANXA1	P04083	373	7.32	30	200	7.92E-16	-1.89	-3.33	PM
Annexin A2	ANXA2	H0YN42	357	8.48	32	268	1.26E-22	-1.91	-1.55	PM
Annexin A2	ANXA2	H0YKV8	892	7.57	13	109	9.98E-07	2.11	ND	PM
Annexin A3	ANXA3	P12429	389	6.09	28	268	1.26E-22	-1.80	-2.66	PM
Annexin A5	ANXA5	D6RBL5	603	4.76	16	111	6.29E-07	1.95	1.98	PM
Apolipoprotein A-I	APOA1	P02647	515	5.26	19	352	5.00E-31	1.70	1.62	ES
ATP synthase subunit alpha, mt	ATP5A1	A8K092	605	5.64	16	173	3.97E-13	ND	1.76	CP
ATP synthase subunit alpha, mt	ATP5A1	AAH08028.2	737	4	10	173	3.97E-13	3.35	3.51	CP
ATP synthase subunit beta	ATP5B	H0YH81	219	4.88	48	409	9.98E-37	-1.62	-1.64	CP
Bestrophin-3	BEST3	NP_001269542	697	7.77	12	39	9.98E+00	1.82	2.13	NP
			671	8.66	14	40	5.00E+00	3.71	3.48	
			648	8.37	15	42	7.92E+00	2.04	1.86	
Calmodulin	CALM1	E7ETZ0	870	3.64	16	63	3.97E-02	1.77	ND	CP
			868	3.59	16	343	3.97E-30	1.65	ND	
			774	3.72	16	367	2.51E-32	1.67	ND	
Centromere protein H	CENPH	B3KVZ3	686	4.81	13	38	1.26E+01	2.67	2.41	NP
Cofilin 1 (Non-muscle)	CFL1	G3V1A4	611	7.14	16	61	6.29E-02	-1.73	-1.70	NP
Elongation factor Tu, mt	TUFM	P49411	262	7.68	41	195	2.51E-15	-2.47	-1.99	CP
			260	7.88	41	338	1.26E-29	-2.21	-1.87	
Enolase 3	ENO3	E5RGZ4	698	4.62	12	63	3.97E-02	1.74	1.51	CP
			235	7.42	45	85	2.51E-04	-1.64	ND	
Ferritin ligt chain	FTL	P02792	572	5.8	18	74	1.26E+01	1.65	2.13	ES
Fibrinogen alpha chain 2	FGA	P02671-2	127	9.09	68	62	9.98E-04	1.94	3.30	ES
			271	8.58	41	72	5.00E-03	-1.83	-2.09	
			377	8.65	30	79	2.51E-12	-1.85	-1.79	
			208	8.67	50	101	5.00E-02	-1.81	-1.74	
			889	7.8	70	165	6.29E-06	1.72	2.91	
Fibrinogen beta chain	FGB	D6REL8	201	7.62	50	64	3.15E-02	-1.74	ND	ES
Fibrinogen beta chain	FGB	P02675	191	6.88	52	107	1.58E-06	-1.92	-1.54	ES
			213	7.66	49	156	1.99E-11	-1.74	ND	
			197	7.48	51	157	1.58E-11	-2.38	-2.29	
			195	7.17	51	163	3.97E-12	-2.07	1.59	
			196	7.02	51	170	7.92E-13	-1.82	-1.62	
			214	8.14	49	212	5.00E-17	-1.70	-1.60	
			202	8.09	50	232	5.00E-19	-2.04	ND	
			200	7.8	50	329	9.98E-29	-1.97	-1.78	
			205	8.5	50	336	1.99E-29	-1.94	-1.69	
Filamin-A	FLNA	P21333	103	6.41	82	238	1.26E-19	-2.11	-1.84	CP
			96	6.07	86	169	9.98E-13	-2.36	-2.11	
Filamin-A	FLNA	Q5HY54	97	6.2	85	164	3.15E-12	-2.03	-1.89	CP
			102	6.68	83	210	7.92E-17	-2.81	-1.99	
			94	5.95	87	238	1.26E-19	-2.95	-2.25	
			100	6.31	83	325	2.51E-28	-1.94	-1.83	
			91	5.81	87	330	7.92E-29	-2.43	-2.01	
			92	6.93	87	384	3.15E-34	-1.68	-1.56	
			93	5.89	87	389	9.98E-35	-2.52	-2.02	
			95	6.81	87	399	9.98E-36	-3.08	-2.15	
Fructose-bisphosphate aldolase	ALDOA	H3BMQ8	353	8.67	32	146	1.99E-10	-1.93	-2.12	CP
Fructose-bisphosphate aldolase	ALDOA	H3BQN4	379	9.32	30	176	1.99E-13	-1.87	-1.87	CP
Gelsolin isoform 2	GSN	P06396-2	104	6.52	82	182	5.00E-14	-2.28	-2.22	ES
Glutathione	GSH	P07203	549	6.94	19	71	6.29E-03	-2.00	-1.89	CP
Glutathione S-transferase Ω 1	GSTO1	P78417	441	7.13	23	296	1.99E-25	-2.09	-1.91	CP
Glyceraldehyde-3-P dehydrogenase	GAPDH	E7EUT4	367	8.45	31	82	5.00E-04	ND	-1.67	CP
			346	8.99	33	164	3.15E-12	-1.76	-2.28	
Haloacid dehalogenase-like hydrolase domain protein 2	HDHD2	K7ER15	431	6.7	24	102	5.00E-06	-1.53	-1.95	Unknown
Heat shock protein 60 kDa, mt	HSPD1	E7EXB4	257	7.96	42	51	6.29E-01	-2.08	-1.72	CP
Heat shock protein 71 kDa	HSPA8	P11142-2	124	5.21	68	325	2.51E-28	-1.82	-1.97	CP
Hemoglobin subunit beta	HBB	P68871	901	8.64	13	254	3.15E-21	2.06	1.87	CP
			685	7.56	13	255	2.51E-21	2.45	3.04	
			905	8.29	13	330	7.92E-29	2.74	2.35	
			904	8.26	13	349	9.98E-31	2.98	2.61	
			902	8.6	13	350	7.92E-31	2.14	1.87	
			903	8.6	13	353	3.97E-31	2.17	1.84	
			690	7.92	13	384	3.15E-34	2.65	2.53	
Heterogeneous nuclear ribonucleoprotein K	HNRNPK	P61978	637	4.35	15	42	5.00E+00	3.13	4.61	CP
Heterogeneous nuclear ribonucleoprotein K	HNRNPK	F8W646	650	7.26	15	42	5.00E+00	ND	-1.85	NP
Heterogeneous nuclear ribonucleoproteins A2/B1	HNRNPA2B1	P22626	372	8.74	31	226	1.99E-18	-1.92	-2.12	NP
Histone H4	HIST1H4A	P62805.2	696	3.77	13	60	7.92E-02	2.80	3.18	NP
			725	7.67	11	70	7.92E-03	2.81	2.23	
			772	5.37	13	193	3.97E-15	1.91	3.03	
			683	5.01	13	242	5.00E-20	5.18	6.07	
Integrin alpha-IIb isoform 3	ITA2B	P08514.3	165	4.19	55	268	7.92E+00	2.07	2.35	PM
Keratin, type I cytoskeletal 10	KRT10	P13645	199	7.3	50	108	1.26E-06	-2.17	-1.65	CP
			524	6.09	28	296	1.99E-25	ND	1.97	
			131	5.35	68	296	1.99E-25	-2.34	-2.56	
Keratin, type I cytoskeletal 9	KRT9	P35527	423	6.91	25	48	1.26E+00	-1.84	-1.73	CP
			81	6.62	94	188	1.26E-14	-3.59	-2.74	
			863	5.47	13	475	2.51E-43	2.60	4.09	
			148	7.38	60	182	5.00E-14	-1.88	-1.56	
Keratin, type II cuticular Hb3	KRT83	P78385	797	5.34	13	165	2.51E-12	3.36	4.72	CP
Keratin, type II cytoskeletal 1	KRT1	P04264	738	3.88	10	96	1.99E-05	5.30	4.23	CP
			582	4.02	17	116	1.99E-07	2.83	3.12	
			796	5.29	13	127	1.58E-08	4.41	5.51	
			533	7.12	19	180	7.92E-14	-1.55	-1.76	
			761	4.82	16	789	9.98E-75	3.66	3.67	
Lactotransferrin Delta	LTF	P02788-2	825	8.65	72	147	1.58E-10	1.59	1.73	CP
LVV-hemorphin-7	HBB	F8W6P5	703	7.72	12	41	6.29E+00	2.20	ND	CP
			867	4.91	0	65	2.51E-02	3.95	4.61	
			804	6.96	10	122	5.00E-08	1.74	1.62	
			879	6.87	11	213	3.97E-17	2.35	1.87	
Mitochondrial carrier homolog 1, isoform 3	MTCH1	Q9NZJ7-3	552	3.85	19	31	6.29E+01	3.02	2.69	CP
	MTCH1	Q9NZJ7-3	666	3.82	14	32	5.00E+01	2.54	2.40	
	MTCH1	Q9NZJ7-3	554	7.1	18	38	1.26E+01	-2.12	-2.27	
	MTCH1	Q9NZJ7-3	583	4.08	17	39	9.98E+00	1.99	2.36	
	MTCH1	Q9NZJ7-3	707	4.58	12	41	6.29E+00	2.10	2.37	
Myeloperoxidase (Fragment)	MPO	P05164-3	170	9.41	55	79	6.29E+00	-1.65	ND	CP
Myeloperoxidase (Fragment)	MPO	J3QSF7	168	8.6	55	107	1.58E-06	-2.25	-1.90	CP
Myeloperoxidase H14	MPO	P05164-2	272	8.7	41	83	3.97E-04	-1.56	-1.69	CP
Myosin light polypeptide 6	MYL6	P60660.2	628	3.79	15	302	5.00E-26	3.74	4.05	CP
			680	7.19	13	421	6.29E-38	1.68	1.69	
Myosin regulatory light 9	MYL9	P24844	612	4.19	16	233	3.97E-19	2.22	2.09	CP
Myotrophin	MTPN	C9JL85	712	5.1	12	146	1.99E-10	2.78	3.36	NP
Nucleosome assembly protein 1-like 1	NAP1L1	F8VRJ2	204	4.29	50	79	9.98E-04	ND	1.62	NP
Fibrinogen alpha isoform 2	FGA	P02671-2	273	8.84	41	79	9.98E-04	1.45	-2.10	ES
Peptidyl-prolyl cis-trans isomerase A	PPIA	P62937	425	8.63	25	110	5.00E+00	2.05	1.81	CP
			630	8.05	15	199	9.98E-16	-2.10	-2.16	
			632	9.02	15	235	2.51E-19	-1.97	-2.05	
Peroxiredoxin-6	PRDX6	P30041	501	6.78	20	237	1.58E-19	-1.94	-1.63	CP
			481	6.81	20	306	1.99E-26	-1.77	-1.96	
Phosphoglycerate kinase 1	PGK1	P00558	300	8.4	38	250	7.92E-21	-1.64	-1.78	CP
POTE ankyrin domain E	POTEE	Q6S8J3	90	5.66	87	135	2.51E-09	-2.31	-2.03	Unknown
POTE ankyrin domain F	POTEF	A5A3E0	261	6.57	41	135	2.51E-09	1.66	2.29	Unknown
Protein FAM212B	FAM212B	Q9NTI7	60	7.09	105	29	9.98E+01	-2.02	-1.78	Unknown
Protein S100-A11	S100A	P31949	878	6.9	10	39	9.98E+00	-2.29	-3.70	CP
			706	6.7	12	260	7.92E-22	1.88	ND	
Protein S100-A6	S100A6	P06703	866	4.88	10	39	9.98E+00	5.11	5.86	CP
			721	3.93	11	68	1.26E-02	2.93	2.89	
Protein S100-A8	S100A8	P05109	809	8.23	11	66	1.99E-02	1.83	ND	CP
Pyruvate kinase	PKM	H3BTN5	167	8.4	55	197	1.58E-15	-2.07	-3.15	CP
Pyruvate kinase M1/M2	PKM	P14618	169	8.76	55	371	6.29E-33	-2.70	-2.12	CP
Ras suppressor protein 1	RSU1	Q15404	465	8.36	22	204	3.15E-16	-1.97	-1.94	CP
Ras-related protein Ral-B	RALB	F8WEQ6	787	5.29	12	35	2.51E+01	2.25	2.88	CP
			713	4.9	12	37	1.58E+01	1.72	2.01	
			718	4.53	11	48	1.26E+00	4.85	5.17	
Regulating synaptic membrane exocytosis protein 2, isoform 4	RIMS2	Q9UQ26-4	845	4.09	68	37	1.58E+01	2.22	2.05	PM
Rho GDP-dissociation inhibitor 2	ARHGDIB	H0YGX7	614	8.69	16	130	7.92E-09	ND	-1.55	CP
Septin-7 isoform 2	SEPT7.	Q16181-2	242	9.3	45	118	1.26E-07	-1.73	ND	CP
SH3 domain-binding glutamic acid- rich-like protein 2	SH3BGRL2	Q9UJC5	704	8.06	12	314	3.15E-27	2.65	2.06	CP
SH3 domain-binding glutamic acid- rich-like protein 3	SH3BGRL3	Q9H299	745	4.2	0	171	6.29E-13	3.11	3.28	NP
			911	4.69	10	354	3.15E-31	2.56	2.72	
Stromal interaction molecule 2	STIM2	Q9P246	344	3.63	33	33	3.97E+01	7.00	5.52	NP
			877	3.8	55	39	9.98E+00	8.00	4.55	
Superoxide dismutase, mt	SOD2	B3KUK2	544	8.41	19	209	9.98E-17	-1.54	-1.65	CP
Thrombospondin-1	THBS1	P07996	732	4.72	11	115	2.51E-07	3.37	3.75	ES
Transcription factor 4	TCF4	H3BME8	241	8.79	45	36	1.99E+01	-2.42	-2.67	NP
Transcriptional repressor YY1	YY1	H0YJV7	568	3.62	18	39	6.29E+00	4.62	3.70	NP
			723	8.67	11	41	5.00E+00	2.15	1.67	
Transketolase	TKT	B4E022	136	8.84	68	189	9.98E-15	1.79	2.67	CP
Tropomyosin 1 alpha	TPM1	D9YZV8	358	4.56	31	515	2.51E-47	-2.03	-2.21	CP
Tropomyosin 3	TPM3	Q5VU59	885	4.37	21	62	5.00E-02	1.57	1.63	CP
			884	4.4	22	421	6.29E-38	1.78	ND	
			142	4.51	63	743	6.29E-38	ND	1.51	
Tropomyosin alpha-4 chain	TPM4	P67936	397	4.01	27	101	6.29E-06	1.54	ND	CP
			782	4.65	27	665	2.51E-47	-2.35	-2.46	
Tropomyosin beta isoform 2	TPM2	P07951-2	324	4.74	36	258	1.26E-21	-1.87	-1.88	CP
Urea transporter 1	SLC14A1	K7EJ54	740	4.86	0	39	9.98E+00	3.55	3.57	PM
UTP-glucose-1-phosphate uridylyltransferase isoform 2	UGP2	Q16851-2	224	8.15	47	85	2.51E-04	-1.98	-2.22	CP
Vimentin	VIM	B0YJC4	540	4.3	19	182	3.97E-16	2.47	2.51	PM
			518	4.29	19	203	1.26E-65	2.12	1.93	
			304	8.69	38	479	5.00E-14	-1.61	-1.79	
			312	8.69	37	632	5.00E-59	-2.07	-2.70	
			307	4.42	37	698	9.98E-44	1.97	1.60	
			385	5.72	29	182	5.00E-14	ND	1.57	
Vinculin	VCL	P18206	63	7.53	99	236	2.51E-04	-1.59	ND	PM
			52	6.39	108	709	9.98E-67	-2.04	-2.33	
			59	6.94	107	1170	7.92E-113	-2.92	-2.75	
Vinculin isoform 1	VCL	P18206-2	89	5.55	88	323	3.97E-28	-1.91	-1.89	PM
			57	6.68	107	1130	7.92E-109	-3.53	-3.87	
			54	6.57	108	1180	7.92E-114	-3.23	-3.91	
			58	6.8	107	1210	7.92E-117	-3.49	-3.31	

The PBMC protein samples from normal/healthy (N/H), chagasic/clinically-asymptomatic (C/A) and chagasic/clinically-symptomatic (C/S) subjects were resolved by 2D-GE approach. Gel images were analyzed with SameSpotst software and normalized spot volumes were used for comparison of C/A(n = 25) or C/S (n = 28) groups with N/H group (n = 30). Proteins spots with ≥ |1.5| fold change (p<0.05) in chagasic subjects were subjected to MALDI-TOF MS/MS analysis. The putative molecular/biological function and cellular location were identified using Ingenuity Pathway Analysis and UniProt software. The e-values present the confidence of protein ID assignment from the MS identifications. Abbreviations: ND: No detectable change, pI: Isoelectric pH, MW: molecular weight, PM: plasma membrane, NP: nucleoplasm, CP: cytoplasm, ES: extracellular space

### IPA network analysis the proteome signature of Chagas disease

We performed IPA analysis to predict the molecular and biological relationship of the differential proteome datasets (Table 2). IPA recognizes all isoforms (e.g. gel-detected pI and size variants of actin, fibrinogen) as the same protein and collapsed the dataset to 82 and 78 differentially abundant proteins in seropositive subjects with no heart disease and those with LV dysfunction, respectively. IPA analysis of the differential proteome datasets predicted an increase in cytoskeletal disassembly and disorganization (z-score: -1.091 to -0.248, S1 Fig), immune cell aggregation (ALB↓, FGA↑, GSN↓, MPO↓, THBS1↑, z-score: 1.521, p value 1.48E-03) and recruitment/activation and migration of immune cells in chagasic (vs. normal) subjects (z-score: 0.501–1.698, p value: 1.94–5.29E-04, S2 Fig), though invasion capacity of cells was decreased in C/S subjects (S2 Fig panel B). Molecular and cellular function annotation of the proteome datasets by IPA predicted a balanced cell proliferation/cell death response in C/A subjects (S3 Fig panel A) while cell death along with inhibition of cell survival was dominantly predicted in PBMCs of C/S subjects (S3 Fig panel B, z-score: 0.858–2.406). IPA also implied a pronounced increase in production of free radicals associated with a decline in scavenging capacity with progressive disease in chagasic subjects (z-score: 1.019 to -1.455, S4 Fig).

The top up-stream molecules predicted to be deregulated and contributing to the differential proteome with disease progression in chagasic subjects included MYC, SP1, MYCN, and growth factor ANGPT2 (z-score -2.266 to -2.190) proteins (S5 Fig).

### MARS modeling of potential protein datasets with high predictive efficacy

We performed MARS analysis to develop a classification model for predicting risk of disease development. MARS is a nonparametric regression procedure that creates models based on piecewise linear regressions. It searches through all predictors to find those most useful for predicting outcomes, and then creates optimal model by a series of regression splines called basis functions [28,29]. For this, MARS uses a two-stage process; first half of the process involves creating an overly large model by adding basis functions that represent either single variable transformations or multivariate interaction terms. In the second stage, MARS deletes basis functions in order of least contribution to the model until the optimum one is reached. End result is a classification model based on single variables and interaction terms which will optimally determine class identity [28,29].

**Fig 5:**
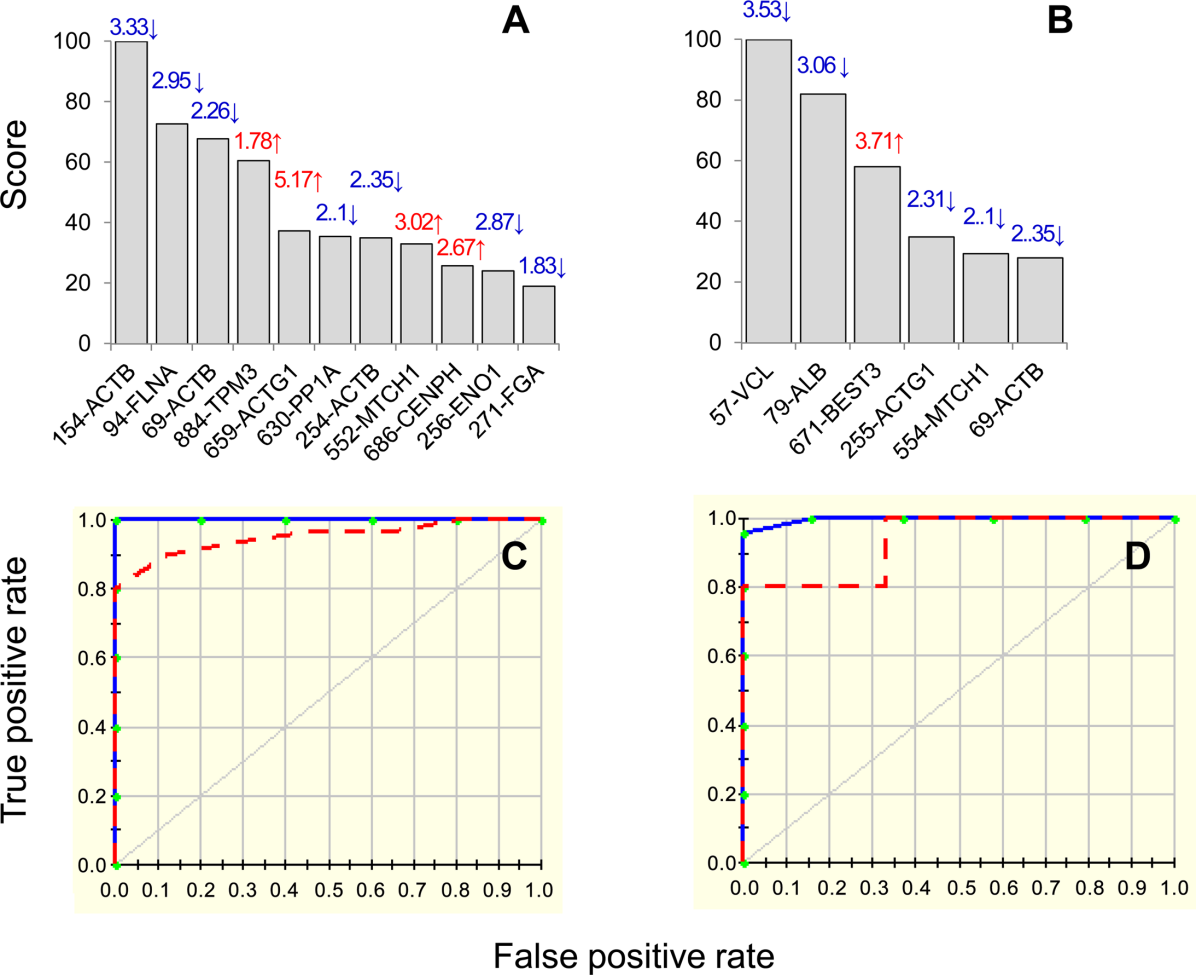
MARS model for classification of seropositive/chagasic subjects. Input to the model were protein spots that were differentially expressed at p<0.001 (with B-H correction) in seropositive, clinically asymptomatic (84 spots, n = 25) subjects with respect to normal/healthy controls (n = 30). We employed 10-fold cross-validation (A&C) and 80% testing / 20% training (C&D) approaches to assess the fit of the model for testing dataset. Shown are the protein spots identified with high ranking (score >20) by cross-validation **(A)** and 80/20 **(B)** approaches for creating the MARS model for classifying C/A subjects from N/H controls. Protein spots in panels A&B are identified as spot #-protein name, and fold change (increase ↑, red; decrease ↓, blue) are plotted on each bar. The ROC curves show the prediction success of the cross-validation **(C)** and 80/20 models **(D)**. Blue curves: training data ((AUC/ROC: 1.00), red curve: testing data (AUC/ROC: 0.96 for CV and 0.933 for 80/20).

Inputs to the model were log2 transformed values for protein spots that were differentially abundant in seropositive/no disease (84 spots, n = 25) and clinically-symptomatic (87 spots, n = 28) groups with respect to normal controls (n = 30) at p<0.001 with B-H correction. We assessed the model accuracy by looking at the prediction success rate and the ROC curves. To address the possible issue of over-fitting the data, we employed two approaches: 1) 10-fold cross validation (CV) allowing same number of maximum basis functions as were the differentially abundant protein spots at p<0.001 (with 1 max interaction term), and 2) testing/training approach in which 80% of the data was utilized for creating the model and the 20% of the remaining data was used to assess the fit of the model for testing dataset. The CV and 80/20 approaches identified 11 and 6 protein spots, respectively, with high importance (score >20, Fig 5A & 5B) for creating the MARS model, detecting differences between the controls and seropositive/no disease subjects. The prediction success showed the CV and 80/20 models fitted perfectly on the training dataset (AUC/ROC: 1.00) and by >93% on the testing dataset (AUC/ROC: 0.96 for CV and 0.933 for 80/20) (Fig 5C & 5D).

**Fig 6:**
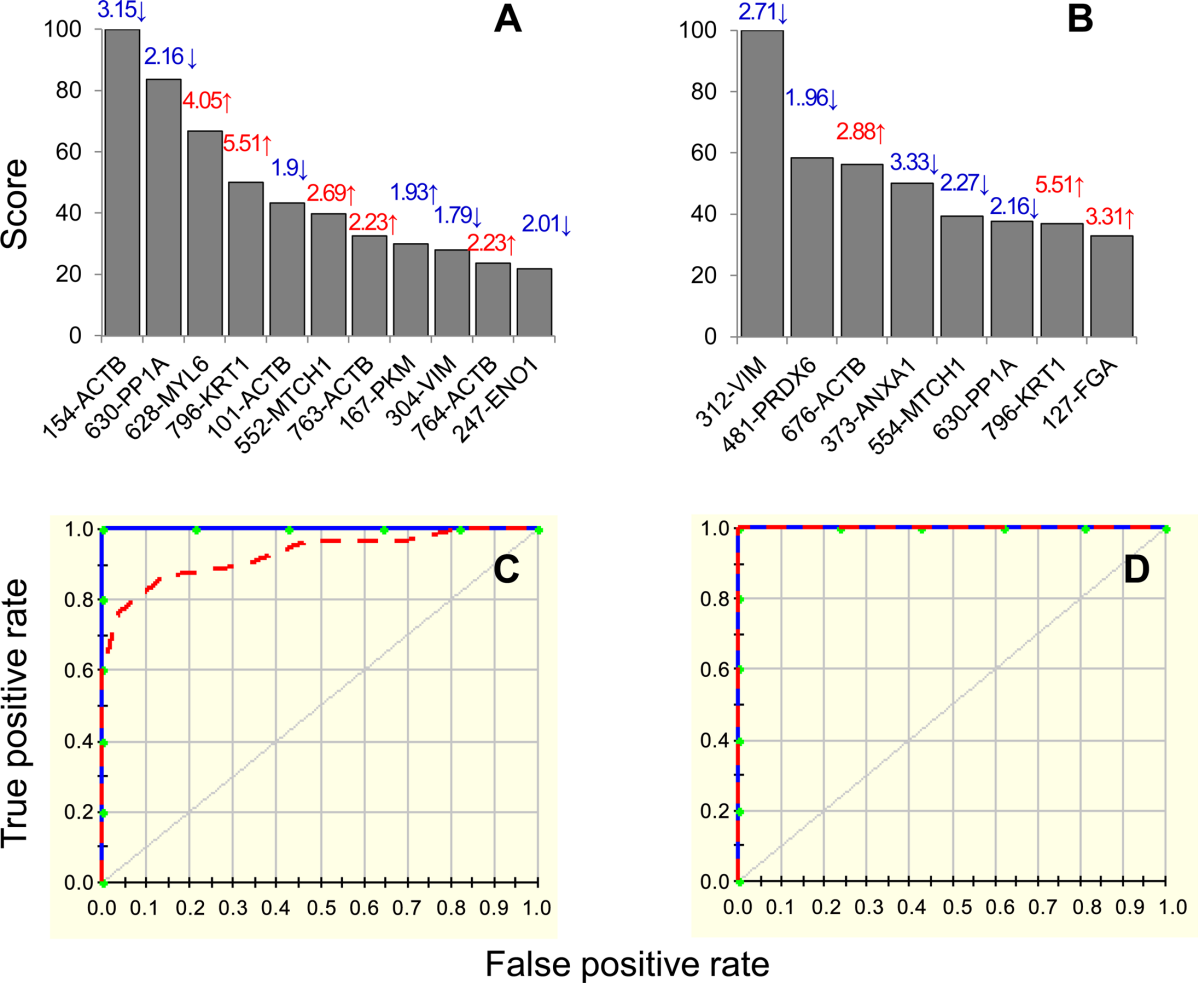
MARS model for classification of chagasic subjects exhibiting clinical disease. Input to the model were protein spots that were differentially expressed at p<0.001 (with B-H correction) in clinically symptomatic (C/S) chagasic subjects (87 spots, n = 25) in comparison to normal/healthy (N/H) controls (n = 30). We employed 10-fold cross-validation (A&C) and 80% testing / 20% training (C&D) approaches to assess the fit of the model for testing dataset. Shown are the protein spots identified with high ranking (score >20) by cross-validation **(A)** and 80/20 **(B)** approaches for creating the MARS model for classifying C/S subjects from N/H subjects. Protein spots in panels A&B are identified as spot #-protein name and fold change (increase ↑, red; decrease ↓, blue) are plotted on each bar. The ROC curves show the prediction success of the cross-validation **(C)** and 80/20 models **(D)**. Blue curves: training data ((AUC/ROC: 1.00), red curve: testing data (AUC/ROC: 0.926 for CV and 1.0 for 80/20).

Likewise, the CV and 80/20 approaches identified 11 and 8 protein spots, respectively, with high importance (score >20, Fig 6A & 6B) for creating the MARS model distinguishing controls from clinically-symptomatic chagasic patients. The prediction success of the CV and 80/20 models were 100% for the training data (AUC/ROC: 1.00). When fitted on testing data, the CV model exhibited very high prediction success (AUC/ROC: 0.926, Fig 6) while the 80/20 model fitted perfectly on the training data (AUC/ROC: 1.00, Fig 6D). These analyses suggested that PBMC changes in the selected protein spots will have high specificity and sensitivity in predicting the disease state in chagasic subjects in comparison to normal/healthy controls.

## Discussion

This study was aimed at assessing the proteomic changes in PBMCs of chagasic subjects grouped as clinically asymptomatic (C/A, n = 25) and clinically symptomatic with heart involvement (C/S, n = 28) in comparison with healthy subjects (n = 30). 2DE/ MALDI-TOF MS analysis identified 213 and 199 protein spots that were differentially abundant in C/A and C/S subjects in comparison to normal/healthy controls (Table 2).

The major cell populations in PBMCs are lymphocytes (B, T and NK cells, ≥70%) and monocytes/macrophages (10–30%). Very few studies have, however, characterized the role of peripheral immune cells in parasite control vs. cardiac pathology in Chagas disease. For example, a recent study noted detection of no NK cells in early infection [30]. In late acute stage of infection, a selective increase in a distinct lineage of NK cells (CD16^+^CD56^–^), as well as a persistent expansion of B cells, possibly indicative of a relationship between B cell activation and a subset of NK cells was noted in humans [30,31]. Others have demonstrated a robust expansion of T cell response in patients with progressive chronic disease though their role in parasite control vs. pathology remains controversial [32–35]. A high frequency of T cells is found in peripheral blood of indeterminate (i.e. C/A) and cardiac (i.e. C/S) patients [35,36], and CD8^+^ granzyme^+^ T cells were the main cell type found in infiltrating infiltrate in the myocardium [37]. However, recent studies have suggested that CD8^+^T cells found in C/A subjects were parasite-antigen specific and functional, while CD8^+^T cells undergoing immunological exhaustion were noted in C/S patients and their lack of activity contributed to the establishment of pathology [38]. A correlation between the production of inflammatory cytokines (IFNγ > IL-10) by CD4^+^ T cells and monocytes of C/S patients, and the production of Th2 cytokine profile (IL-10 and IL-4) by the same cells of C/A patients is also shown [39,40]. These studies tend to conclude that functional capacity of T cells along with anti-inflammatory activation of monocytes determines the control of parasite and clinically asymptomatic state in chagasic individuals while functionally incapable T cells and consistent proinflammatory activation of monocytes contributes to chronic, clinically symptomatic disease.

IPA analysis of the proteome datasets in this study suggested that differential migration and/or invasion capacity of immune cells may also contribute to host’s ability to control *T*. *cruzi* and enter C/A vs C/S stage. An increase in cellular disassembly and disorganization associated with disruption of filaments that is central to remodeling of the cytoskeleton and modulation of cell shape for migration was observed in PBMCs of all chagasic patients (S1 Fig). Specifically, the expression profile of Ca^2+^-dependent phospholipid-binding members of the annexin family that possess phospholipase A2 inhibitory activity [41], vimentin and actin isoforms (ACTB, ACTG) that are the cytoskeletal component responsible for maintaining cell integrity and are mediators of internal cell motility [42] and filamin A (FLNA) that interacts with several molecules (e.g. integrins) to regulate the actin cytoskeleton organization [43] were all altered in PBMCs of chagasic subjects. However, the expression levels of small G proteins (Rab14, RAP1B) that regulate membrane trafficking across golgi and endosomal compartments [44,45] and of Rab13 that controls junctional development by directly binding to F actin and modifying actin cytoskeletal reorganization [46] and cell spreading via filamins [47] were increased and decreased in C/A and C/S subjects, respectively, and might have played an important role in determining the extent of immune cell migration in C/A versus C/S chagasic subjects. Consistent with this, all seropositive chagasic subjects exhibited an expression profile indicative of increase in migration of phagocytes and leukocytes (S3 Fig), though a small subset of molecules identified to be linked to invasion process (11 molecules, z score: -2.032, p value: 1.43E-03; ANXA1↓, ANXA2↓, FLNA↓, GSN↓, LTF↑, PKM↓, S100A6↑, SOD2↓, THBS1↑, VIM↓, YY1↑, S3 Fig panel B) were decreased in C/S subjects, thus suggesting that functional lymphocytes may be mobilized in periphery but not able to access and kill tissue parasites.

What might be the source of low-grade antigenic stimulus that results in persistence of immune cells and whether these surviving immune cells are functional in the context of parasite control is not entirely clear. Some investigators have argued that it is the long-term persistence of parasitic antigens that result in exhaustion of the functional T cell compartment [48,49]. The authors noted the frequency of parasite-specific functional CD4^+^ and CD8^+^ T cells decreased with more severe stages of clinical disease in human patients, and the T cells that persisted in chronically infected individuals were not metabolically or functionally active and exhibited the phenotypic characteristics of senescence [48,49]. Our data showed an increase in free radical synthesis and a decline in free radical catabolism and scavenging capacity in infected individuals that exhibited more pronounced disease state (S4 Fig, panel B). We and others have shown that oxidative stress is persistent in chronically-infected chagasic animals and patients [14,17,50,51], and oxidized cardiac proteins serve as neo-antigens and recognized by antibody response in chagasic mice and patients [25]. Thus, it is also possible that self-proteins that are oxidized due to persistence of oxidative stress serve as the source of antigenic stimulus for a low-grade but persistent activation of immune cells in chagasic host. The two hypotheses, i.e., parasite or self-antigens contributing to persistence of non-functional, senescent immune cells are not mutually exclusive and together explain why the persistent chronic inflammation is of pathological importance in Chagas disease.

The gene expression studies using global and custom arrays have shown the mitochondrial function-related gene expression is decreased in experimental models of *T*. *cruzi* infection and in the cardiac biopsies of chagasic patients [52–55]. A loss in the activity of mitochondrial respiratory complexes (I and III) was also noted in cardiac biopsies of chagasic rodents [14,56] and peripheral blood of human patients [17] that correlated with decreased coupled respiration and ATP generation [50,57]. In this study, PBMCs of chagasic patients showed protein expression pattern indicative of inhibition of glycolysis/gluconeogenesis (↓PKM, ↓GAPDH, ↓ENO1, ↓ADLOA, and ↓PGK1). The abundance of ATP5A1 that contributes to oxidative phosphorylation and ATP synthesis was counter-effected by abundance of MTCH1 that is localized to the mitochondrion inner membrane and induces Bax- and Bak- independent apoptosis [58,59] in chagasic PBMCs. Further, all isoforms of TUFM that participate in protein translation in mitochondria were decreased in chagasic PBMCs. Mutations in TUFM are shown to contribute to oxidative phosphorylation inefficiency and lactic acidosis in infantile encephalopathy [60]. These data provide a novel clue, and suggest that decreased translation and/or transport of mitochondria-targeted proteins affecting the functional assembly of electron transport chain complexes might play a major role in mitochondrial energy deficiency during progressive Chagas disease.

The top upstream regulators, MYC/MYCN and SP1 were predicted to be inhibited (z-score: < 2, p<0.001, all), and identified as common link contributing to expression profile of protein datasets related to metabolism, cell death/cell proliferation, ROS scavenging and cytoskeletal remodeling in chagasic subjects. MYC and MYCN are very strong proto-oncogenes that play a role in cell cycle, apoptosis and cellular transformation through diverse mechanisms. Recently, MYC has been reported to induce accumulation of DNA oxidative adducts and impair cell cycle regulatory capacity which potentially can increase the genomic instability and provide an environment conducive to growth of the cancer cells [61]. Others have shown MYC-dependent-ROS increase induced cell death [62]. Whether MYC-induced ROS contribute to tumorigenesis in human cells is not clearly demonstrated; however, in the context of chagasic subjects, our study suggests that the inhibition of MYC was likely an adaptive response to control pathological outcomes related to uncontrolled ROS production and immune cell proliferation. Indeed as early as 1992, a selective reduction of c-myc and c-fos mRNAs in association with the severe suppression of the IL-2 gene in lymphoid of mice infected by *T*. *cruzi* was noted [63]. Like MYC, SP1 transcription factor also modulates the expression of genes involved in cell division, apoptosis, and immune responses. Post-translational modifications of SP1 are suggested to alter its DNA binding and transactivation activity and thereby affect the transcriptional activity [64]. Up regulation of SP1 is shown to be tumorigenic and its reduction was found to be neuroprotective in *in vitro* and *in vivo* models of Huntington’s disease [65]. PARP-1, a member of the poly (ADP-ribose) polymerase family, produces poly(ADP-ribose) units (PAR) [66] and PAR modifications of SP1 suppressed its DNA-binding properties [67]. We have shown hyperactivation of PARP-1 stimulated by oxidative DNA damage in cardiomyocytes infected by *T*. *cruzi* [68]. How cross-talk of PARP-1 and SP1 determines the expression and transcriptional function of SP1 in the context of chronic chagasic cardiomyopathy remains to be elucidated in forthcoming studies.

In summary, this study demonstrates that unbiased proteomic analysis of PBMCs in a discovery mode is useful in enhancing our knowledge of the pathomechanisms that determine predisposition to and progression of clinically symptomatic Chagas disease. By employing a 2DE and MALDI-TOF/MS approach for developing the PBMC proteome signature of chagasic subjects, we have identified the possible pathologic mechanisms in disease progression would involve host’s inability to recruit immune cells, scavenge free radicals, and prevent cell death. MYC/SP1 transcription factors that regulate hypoxia and inflammatory stress were predicted to be key targets for controlling chagasic pathology. MARS-modeling identified a panel of protein spots that if monitored in infected individuals, will have >93% success in predicting risk of clinical disease development. Our results provide an impetus for further studies in a second independent cohort of patients for confirming the diagnostic potential of suggested panel of proteins.

## Supporting Information

S1 Fig Molecular/function networks of cytoplasmic/cytoskeletal re-organization during Chagas disease. PBMC proteome of chagasic subjects that were clinically asymptomatic (C/A, n = 25) or clinically symptomatic (C/S, n = 28) with cardiac involvement was compared with the PBMC proteome of normal/healthy (N/H, n = 30) individuals, and protein spots that were differentially abundant in chagasic subjects with respect to N/H controls (p<0.05) were identified by mass spectrometry, as described in Materials and Methods. The differential PBMC proteome datasets (Table 2) were submitted to Ingenuity Pathway Analysis (IPA). Shown is molecular and cellular function network indicative of disorganization of cytoplasm and cytoskeleton in C/A **(A)** and C/S **(B)** chagasic subjects. In all figures, intensity of red and green colors shows the extent of increase and decrease in protein abundance, respectively, in chagasic individuals. Gray and yellow lines indicate putative effect not predicted and findings inconsistent with state of downstream molecule, respectively. Brown node/lines and blue node/lines show predicted activation and inhibition, respectively, of a pathway.
(TIF)

S2 Fig Molecular/function networks indicative of migration of cells in chagasic patients. Shown is molecular and cellular function network of migration of cells including leukocyte and phagocyte population of cells in C/A **(A)** and C/S **(B)** chagasic subjects; developed by IPA analysis of differential PBMC proteome dataset (Table 2). Note the predicted inhibition of cell invasion pathway is in C/S subjects in panel B.
(TIF)

S3 Fig Molecular and cellular function networks of cell death and cell proliferation with progressive Chagas disease. Shown is molecular and cellular function network of cell death/cell proliferation response in C/A subjects **(A)** and cell death/cell survival response in C/S subjects **(B)**; developed by IPA analysis of differential PBMC proteome dataset (Table 2). Note the predicted inhibition of cell survival in C/S subjects in panel B.
(TIF)

S4 Fig Differentially abundant protein datasets indicative of generation and scavenging of ROS in Chagas disease. Shown is molecular and cellular function network of ROS production and scavenging in C/A **(A)** and C/S **(B)** chagasic subjects); developed by IPA analysis of differential PBMC proteome dataset (Table 2). Note the host’s capacity to metabolize ROS was predicted to be down regulated in C/S subjects (panel B).
(TIF)

S5 Fig Top regulatory molecules linked to disease progression in chagasic subjects. Shown are top regulatory molecules, MYC, MYCN, SP1 in C/A subjects **(A)** and ANGPT2, MYC, SP14 in C/S subjects **(B)** that were potentially disturbed and responsible for alterations in the proteome profile of chagasic subjects with respect to N/H controls.
(TIF)
