# Supplementary figures and images for: Changes in Proteome Profile of Peripheral Blood Mononuclear Cells in Chronic Chagas Disease
Source: PLoS Negl Trop Dis. 2016 Feb 26;10(2):e0004490. doi: 10.1371/journal.pntd.0004490 (PMC4769231; doi:10.1371/journal.pntd.0004490)

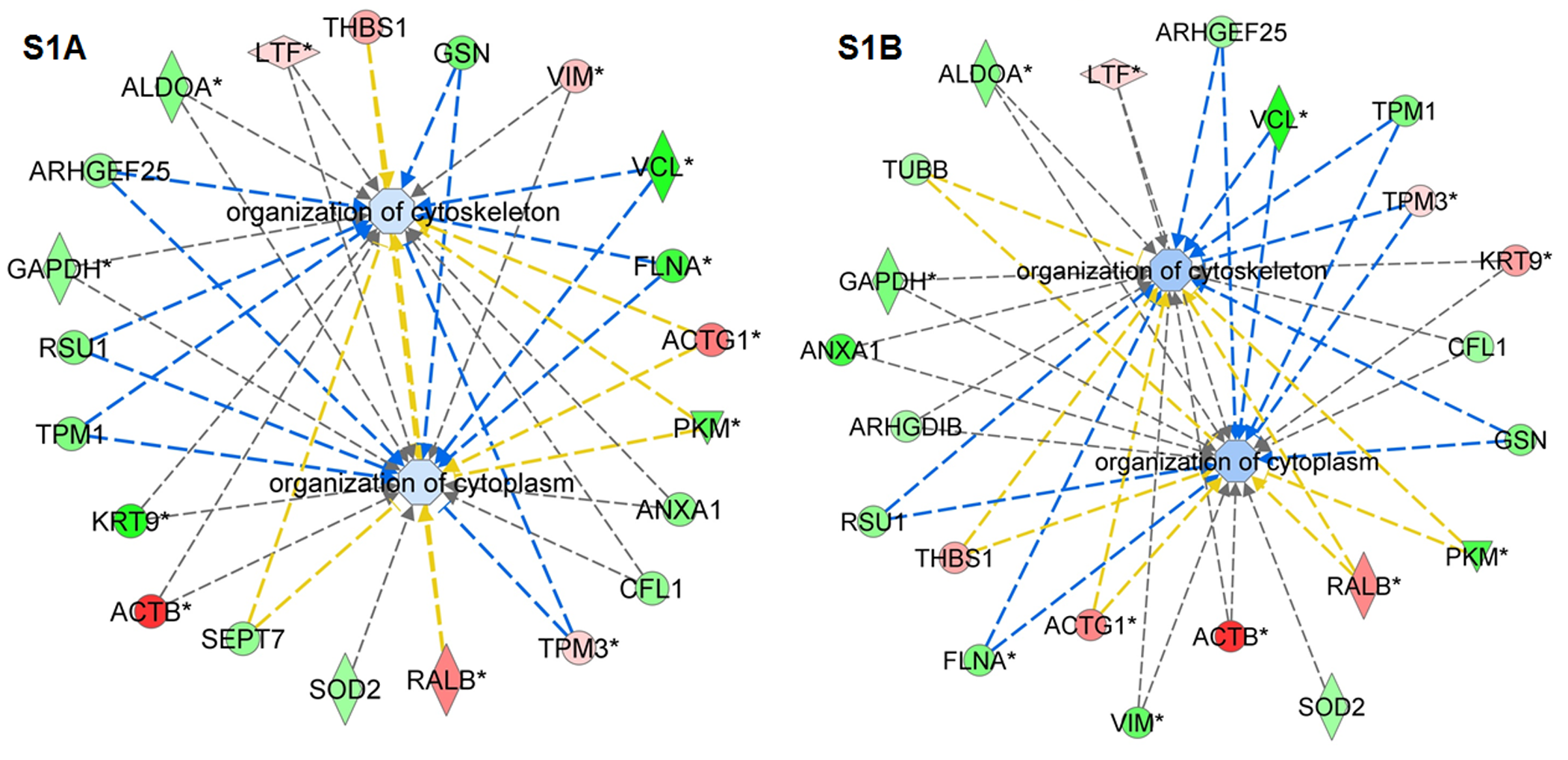

Supplement: S1 Fig — PBMC proteome of chagasic subjects that were clinically asymptomatic (C/A, n = 25) or clinically symptomatic (C/S, n = 28) with cardiac involvement was compared with the PBMC proteome of normal/healthy (N/H, n = 30) individuals, and protein spots that were differentially abundant in chagasic subjects with respect to N/H controls (p<0.05) were identified by mass spectrometry, as described in Materials and Methods. The differential PBMC proteome datasets (Table 2) were submitted to Ingenuity Pathway Analysis (IPA). Shown is molecular and cellular function network indicative of disorganization of cytoplasm and cytoskeleton in C/A (A) and C/S (B) chagasic subjects. In all figures, intensity of red and green colors shows the extent of increase and decrease in protein abundance, respectively, in chagasic individuals. Gray and yellow lines indicate putative effect not predicted and findings inconsistent with state of downstream molecule, respectively. Brown node/lines and blue node/lines show predicted activation and inhibition, respectively, of a pathway. (TIF) [file pntd.0004490.s001.tif]

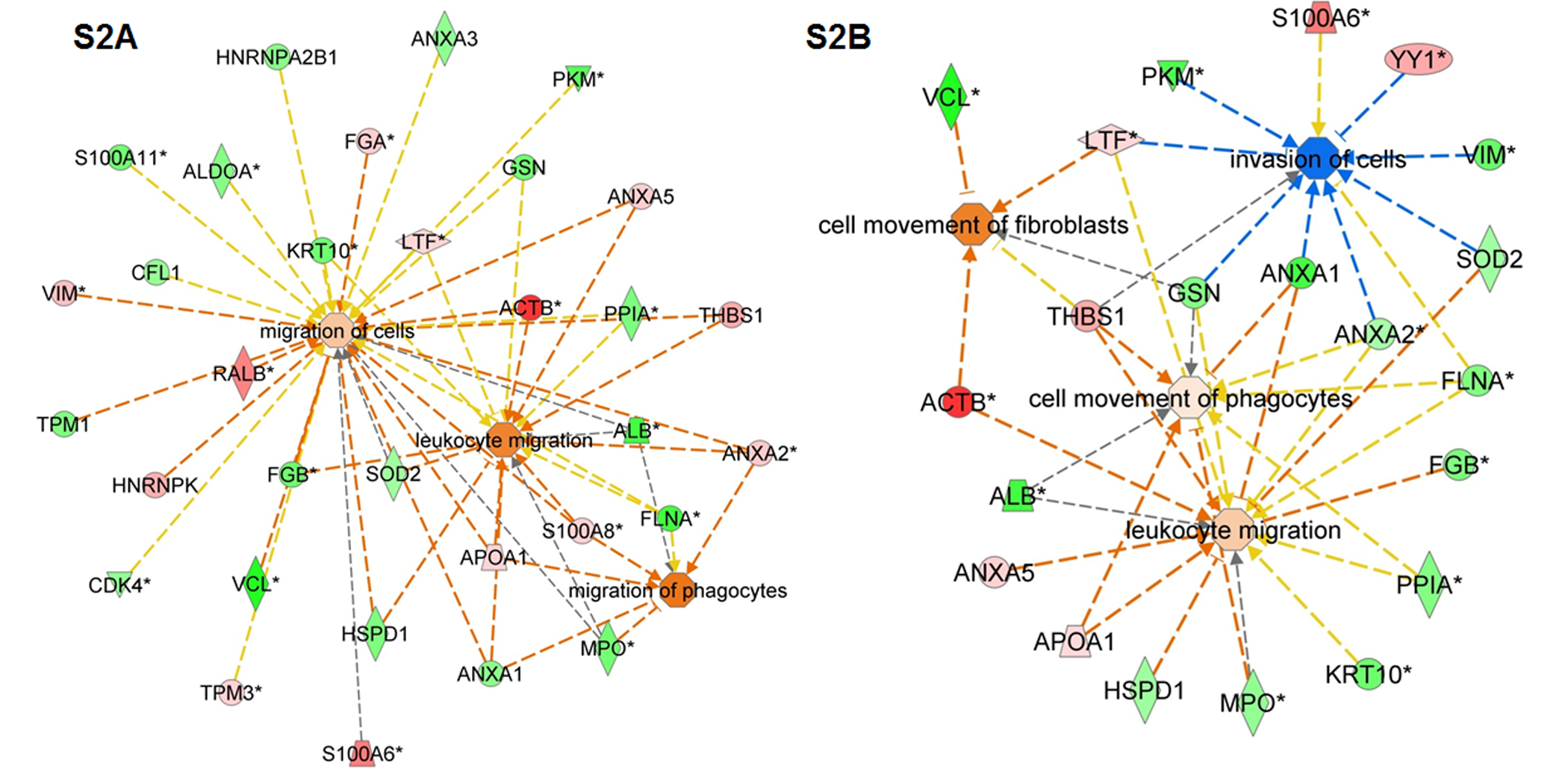

Supplement: S2 Fig — Shown is molecular and cellular function network of migration of cells including leukocyte and phagocyte population of cells in C/A (A) and C/S (B) chagasic subjects; developed by IPA analysis of differential PBMC proteome dataset (Table 2). Note the predicted inhibition of cell invasion pathway is in C/S subjects in panel B. (TIF) [file pntd.0004490.s002.tif]

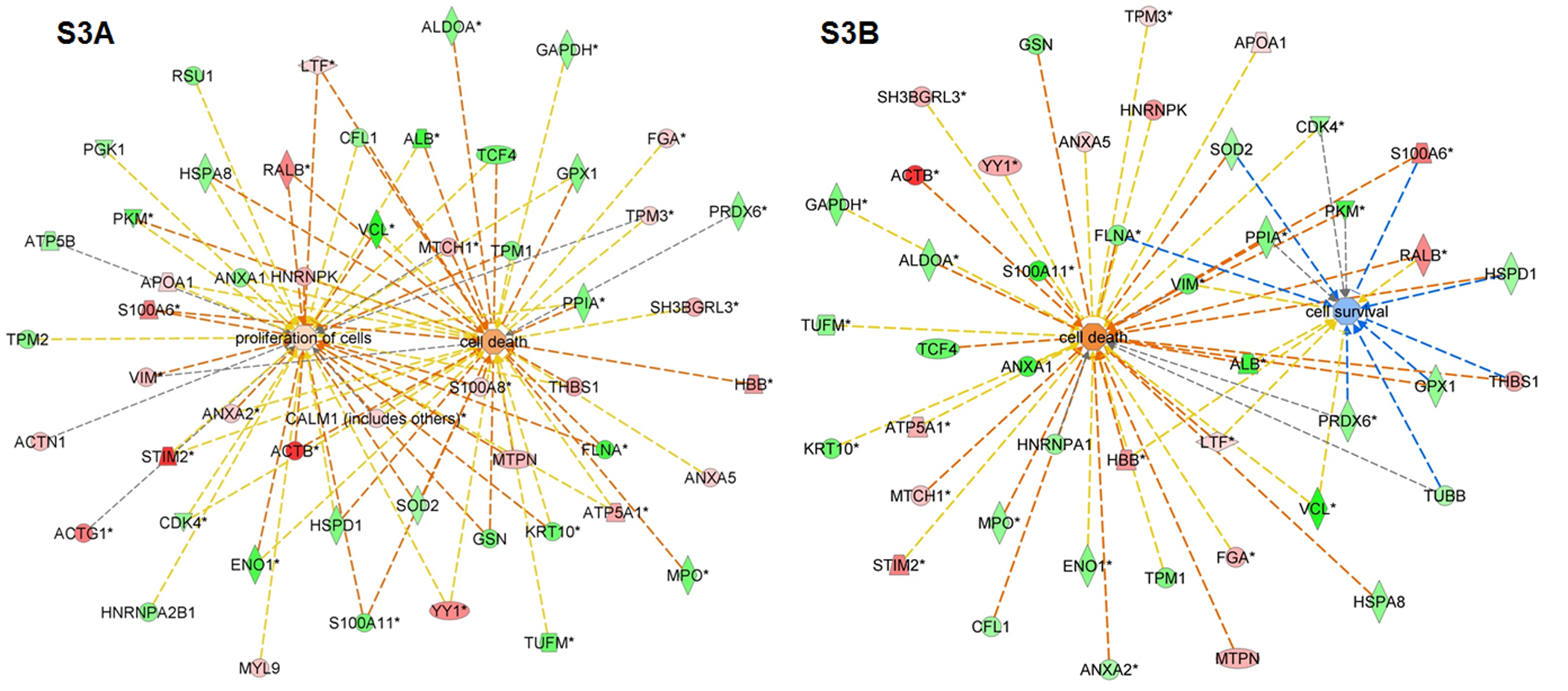

Supplement: S3 Fig — Shown is molecular and cellular function network of cell death/cell proliferation response in C/A subjects (A) and cell death/cell survival response in C/S subjects (B); developed by IPA analysis of differential PBMC proteome dataset (Table 2). Note the predicted inhibition of cell survival in C/S subjects in panel B. (TIF) [file pntd.0004490.s003.tif]

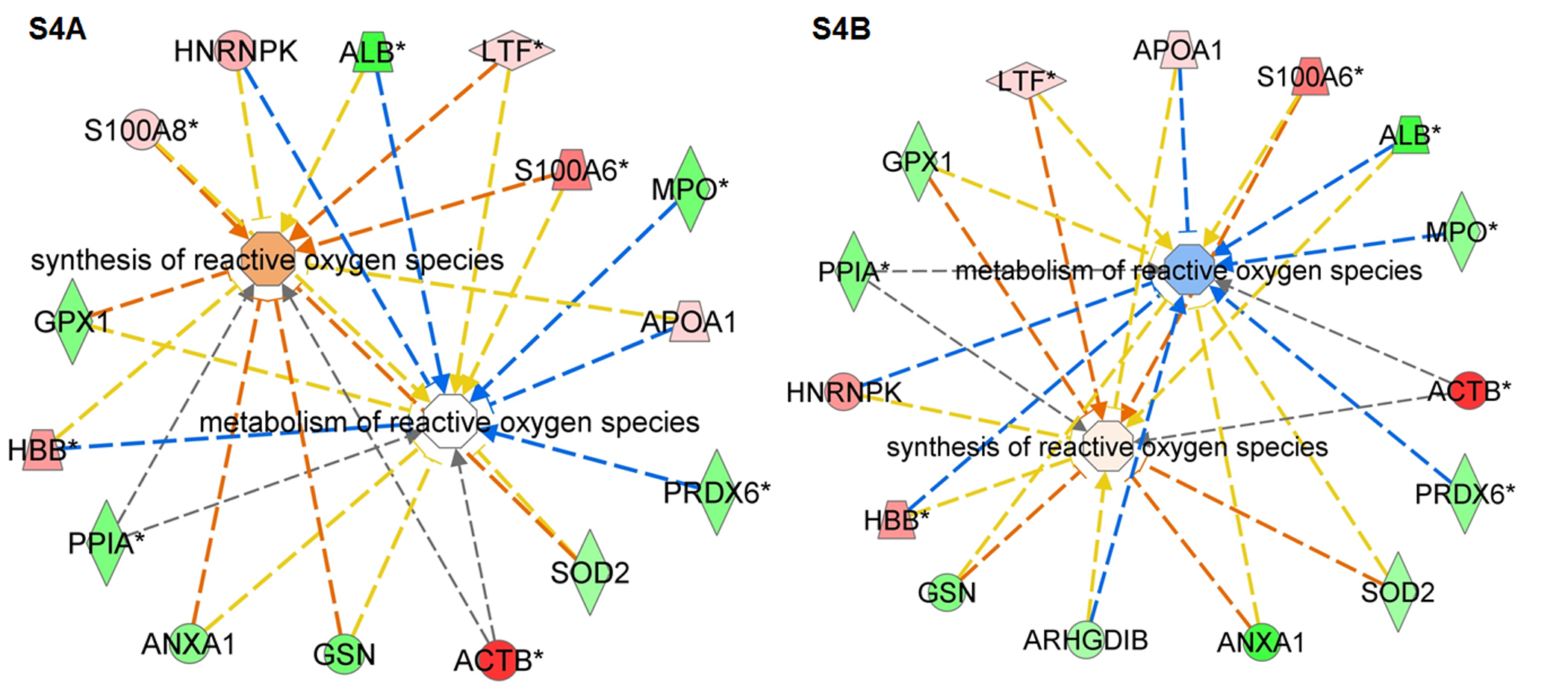

Supplement: S4 Fig — Shown is molecular and cellular function network of ROS production and scavenging in C/A (A) and C/S (B) chagasic subjects); developed by IPA analysis of differential PBMC proteome dataset (Table 2). Note the host’s capacity to metabolize ROS was predicted to be down regulated in C/S subjects (panel B). (TIF) [file pntd.0004490.s004.tif]

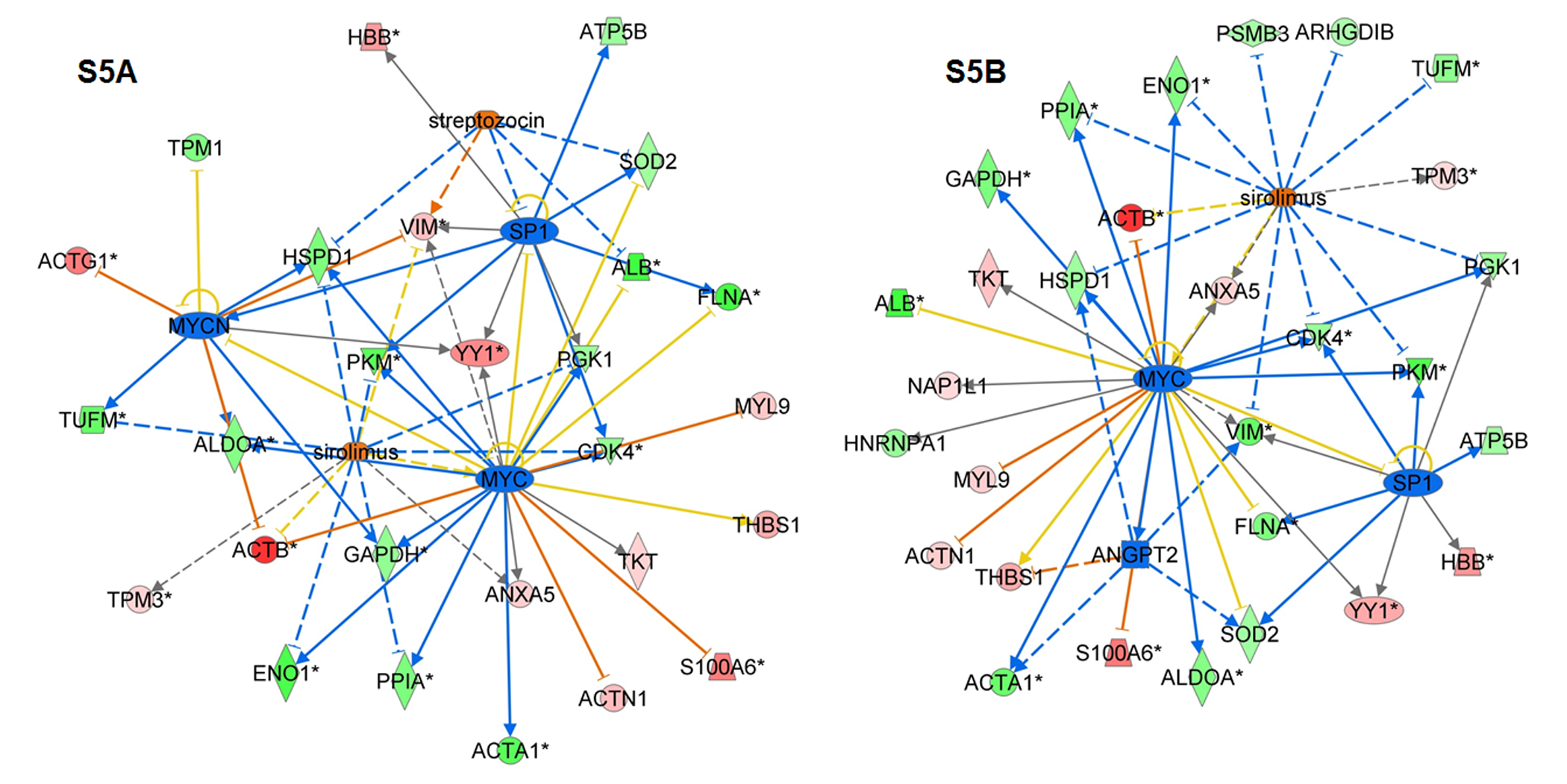

Supplement: S5 Fig — Shown are top regulatory molecules, MYC, MYCN, SP1 in C/A subjects (A) and ANGPT2, MYC, SP14 in C/S subjects (B) that were potentially disturbed and responsible for alterations in the proteome profile of chagasic subjects with respect to N/H controls. (TIF) [file pntd.0004490.s005.tif]
